# Dissolvable microneedle-assisted transdermal administration of diacerein nanoparticles achieved satisfactory therapeutic effects in tendon-bone insertion repair by reducing oxidative stress and inflammation

**DOI:** 10.1016/j.mtbio.2025.101999

**Published:** 2025-06-18

**Authors:** Jie Sun, Qing Zhong Chen, Ai Zi Hong, Fei Ju, Hao Liang Wang, Bo Zhang, Wang Liu, Yu Cheng Sun, Jun Tan, Qian Qian Yang, You Lang Zhou

**Affiliations:** Hand Surgery Research Center, Research Center of Clinical Medicine, Affiliated Hospital of Nantong University, Medical School of Nantong University, Nantong, 226001, China

**Keywords:** Tendon-bone insertion injury, ROS, Macrophages, Dissolvable microneedles, Diacerein

## Abstract

Tendon-bone insertion injuries are frequently encountered in sports medicine clinics, with the healing process posing a significant challenge within this field. When it comes to non-surgical interventions for such injuries, the use of local drug injections may carry the risk of damaging peripheral nerves and blood vessels. In this study, a simple dissolvable microneedle (DMN) patch loaded with diacerein (Dia) nanoparticles (DMN/PLGA@Dia) is established to achieve a constant delivery of Dia for enhancing tendon-bone healing. *In vitro* experiments demonstrate that Dia alleviates oxidative stress, reduces apoptosis, and prevents senescence in tendon cells, while also polarizing macrophages to the M2 phenotype. In a rat model of Achilles tendon-bone insertion injury, the application of DMN/PLGA@Dia enhances the regeneration of the tendon-bone insertion by reducing reactive oxygen species (ROS) levels and shifting macrophage polarization from M1 to M2. Additionally, DMN/PLGA@Dia improves functional recovery, as evidenced by biomechanical and gait analyses. These findings suggest that DMN/PLGA@Dia is capable of promoting tendon-bone insertion repair by alleviating oxidative stress and regulating the immune microenvironment strategies. Simple DMN/PLGA@Dia may be an effective and safe nanomedicine delivery system for tendon-bone insertion repair.

## Highlights

• A dissolvable microneedle (DMN) patch with Diacerein nanoparticles (DMN/PLGA@Dia) enabled sustained Dia delivery to improve tendon-bone healing.
• Dia can alleviate oxidative stress damage in tendon cells and convert macrophages into M2 type.
• Our research findings may be applicable to patients with tendon-bone injuries who choose conservative treatment in clinical practice.

## Graphical abstract

**Figure:**
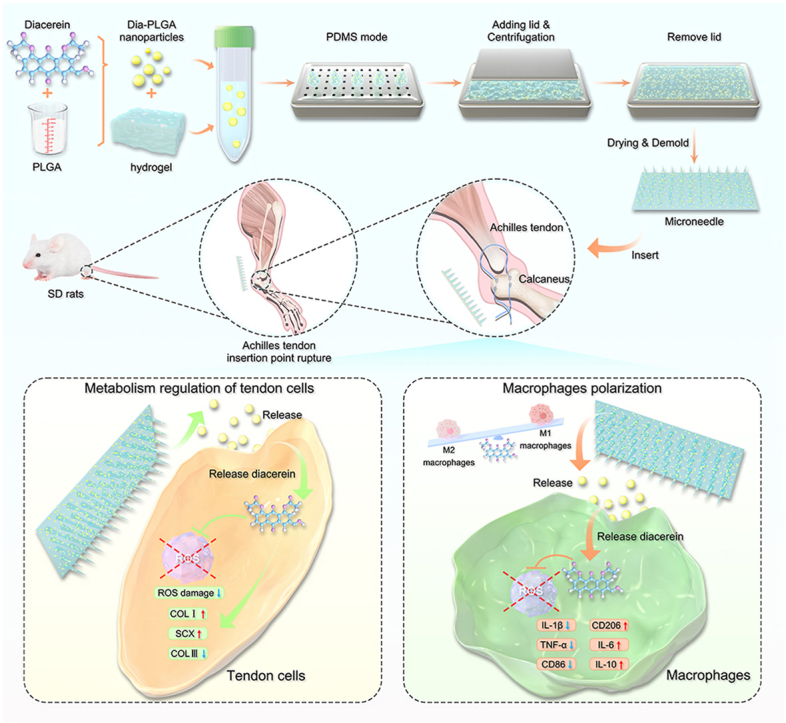
Schematic diagram of the preparation process for the DMN/PLGA@Dia and treatment mechanism for tendon-bone insertion healing by alleviating oxidative stress and regulating the immune microenvironment strategies.

## 1 Introduction

The tendon-bone insertion site constitutes a sophisticated hierarchical tissue, encompassing the pivotal structures of the tendon-bone insertion [1]. Tendon-bone insertion includes the tendon, non-calcified fibrocartilage, calcified fibrocartilage, and bone. Prior studies have indicated that the prevalence of isolated rotator cuff injuries varies between 19.0% and 32.0% [2]. Annually in the US, over 250,000 surgeries for rotator cuff repairs are conducted, with the recurrence rate varying between 11.0% and 94.0% after two years [3]. Approximately half of the patients suffer from enduring post-surgical discomfort that defies alleviation. Even with the swift advancements in surgical methodologies in recent times, the restored tissue seldom recurs to its original, meticulously arranged histological gradient, particularly in the characteristic fibrocartilage structures. Currently, no viable non-surgical treatments exist for addressing tendon and bone injuries. The administration of local injections is fraught with the danger of causing damage to peripheral nerves and blood vessels. The process of healing the insertion point where tendons meet bones persists as a significant challenge within the domain of sports medicine [4].

M1 macrophages serve as the principal orchestrators of inflammation, representing the predominant macrophage subset at the inception of tendon-bone insertion injuries. They are pivotal in the secretion of pro-inflammatory cytokines such as IL-1β and TNF-α, and also contribute to the production of reactive oxygen species (ROS). These actions intensify the inflammatory cascade and foster a pro-inflammatory immune milieu [[5], [6], [7]]. ROS, resultant from oxidative phosphorylation, have demonstrated a role in the healing of tendons [8]. The excessive accumulation of ROS has the potential to trigger tenocyte apoptosis, accelerate collagen degradation, inhibit tenogenic differentiation, and promote the polarization of M1-type macrophages [9]. Recent clinical investigations have indicated that elevated concentrations of superoxide-generated oxidative stress are linked to the recurrence of tears following arthroscopic rotator cuff surgical interventions [10]. The interplay between ROS and M1 macrophages creates a deleterious cycle that significantly contributes to the poor healing of tendon-bone insertions. Under such circumstances, enhancing the proportion of M2 macrophages and inhibiting ROS is crucial for fostering the repair process of tendon-bone junctions.

Diacerein (Dia) serves as a therapeutic agent for the management of osteoarthritis, exhibiting symptomatic relief over time. Its active derivative, Rhein, underscores its efficacy. This non-steroidal anti-inflammatory drug boasts distinctive pharmacological attributes, including potent anti-oxidant and anti-apoptotic effects [11]. It has additionally demonstrated the ability to suppress interleukin-1 activity [12]. In addition to its curative impact on osteoarthritis, Dia has found expanded applications in the treatment of various other conditions in recent times [[13], [14], [15], [16], [17]]. For instance, Mansoure and colleagues have demonstrated that Dia exerted a dose-dependent attenuation against diclofenac-induced acute renal injury in rats, harnessing its potent antioxidative, anti-inflammatory, necroprotective, and apoptosis-inhibitory properties by modulating the SIRT1/HIF-1α/NF-κB and SIRT1/p53 signaling pathways [13]. Refaie notes that Dia successfully counteractes the alterations associated with diabetic cardiomyopathy, attributed to its noted anti-inflammatory, antioxidant, and anti-apoptotic effects, alongside its ability to modulate blood glucose levels [14]. Abdel-Aziz and colleagues discovered that the pretreatment with Dia exhibits potential in reversing the adverse impacts of pulmonary fibrosis. This is indicated by the diminished oxidative stress and pulmonary inflammation, achieved through the downregulation of the TLR4/NF-κB signaling pathway [15]. Given that Dia can suppress the expression of the inflammatory cytokine IL-1β and inhibit ROS, it may prove beneficial for the treatment of tendon-bone healing.

Recently, dissolving microneedles (DMNs) have been revealed to be an effective drug delivery vehicle, while also minimizing the required dose across localization [18,19]. DMNs can penetrate the epidermis and reach the internal tissues in a painless, non-invasive, and non-infectious manner [20]. However, the stability, mechanical strength and drug loading capacity of DMNs are principal problems [21]. In this scenario, incorporating poly (lactic-co-glycolic acid) (PLGA) nanoparticles can enhance the rigidity of microneedles, thereby promoting a sustained drug release [22,23].

**Scheme 1:**
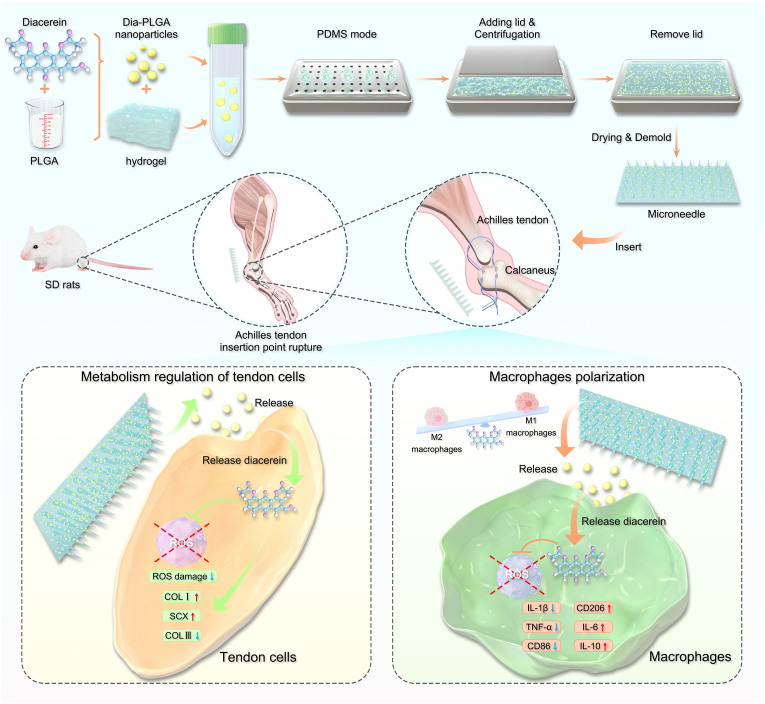
Schematic diagram of the preparation process for the DMN/PLGA@Dia and treatment mechanism by alleviating oxidative stress and regulating the immune microenvironment strategies.

In this study, a type of Dia-loaded nanoparticles, based on ROS scavenging and anti-inflammatory strategies, is developed and subsequently mixed with hydrogel to produce simple microneedles, termed DMN/PLGA@Dia. Hydrogels and PLGA nanoparticles have been confirmed as biologically safe through extensive prior research [24,25]. Scheme 1 presents our research opinions and methods. Local injections of drugs for tendon-bone injuries carry risks such as nerve and vessel damage, while DMNs offer a painless, non-invasive alternative, penetrating the epidermis to deliver drugs directly to the affected area without systemic side effects. This approach not only enhances drug stability and loading capacity but also minimizes the risk of complications associated with traditional injections. By encapsulating Dia in PLGA nanoparticles within DMNs, we achieved sustained and localized delivery of Dia. Dia can effectively alleviate oxidative stress, reduce inflammation, and reconfigure the immune microenvironment after tendon-bone insertion injury. Specifically, our *in vitro* and *in vivo* experiments have displayed that Dia can rebuild the tendon specific differentiation potential of tendon cells under oxidative stress conditions. Concurrently, the pathological mechanisms of cellular senescence and programmed cell death are mitigated in unison. Within the immune chamber, the inflammatory signals are mitigated, leading to a diminished presence of pro-inflammatory cytokines and a concurrent rise in the levels of anti-inflammatory cytokines. Furthermore, Dia has the capability to polarize macrophages towards the M2 phenotype, thereby creating an advantageous environment for tissue regeneration and remodeling. These findings suggest that the DMN/PLGA@Dia complex can effectively facilitate the repair of tendon-bone insertions by mitigating oxidative stress and modulating the immune microenvironment. Furthermore, it has the potential to disrupt the detrimental cycle of ROS accumulation and M1 macrophage polarization following injury.

## 2 Materials and methods

### 2.1 Data processing and functional annotation

Data pertaining to gene expression patterns linked to tendon-to-bone junction injuries are sourced from the Gene Expression Omnibus database of the National Center for Biotechnology Information. Specifically, the dataset GSE103266 is extracted and subjected to a series of computational transformations: background adjustment, standardization, and logarithmic scaling, all facilitated by the Limma R package. The process of identifying differentially expressed genes (DEGs) is guided by stringent thresholds, with P < 0.05 and log2 fold change (logFC) ≥1. Subsequently, functional and pathway enrichments are explored through GO and Kyoto Encyclopedia of Genes and Genomes (KEGG) analyses, executed with the clusterProfiler.

### 2.2 Construction of PPI network

The STRING database serves as a web-based analytical resource for examining reported proteins and forecasting PPI networks. It encompasses both direct and indirect relationships among proteins, as well as their functional associations (https://string-db.org/) [26]. For DEGs that achieve a composite score exceeding 0.4, they are fed into the STRING platform. Then, Cytoscape is employed to visualize and construct the PPI network.

### 2.3 Preparation and characterization of drug-encapsulated nanoparticles

Nanoparticles encapsulating Dia are synthesized via the double emulsion solvent evaporation method. Initially, a 200 μL of a Dia-DMSO solution (10 mg/mL) is combined with 1 mL of a dichloromethane containing 100 mg of PLGA polymer (with a lactic to glycolic acid ratio of 65:35 and a molecular weight range of 40,000–75,000). The mixture is emulsification in 3 mL of a 7% PVA (poly (vinyl alcohol)) solution, which possesses a molecular weight of 14,160, utilizing an ultrasonic homogenizer for 30 s, all while being maintained in an ice bath. Afterwards, the emulsion is moved to 50 mL of 1% PVA solution and sonicated for another 60 s. The mixture is accordingly stirred for a minimum of 24 h at room temperature to assure complete evaporation of dichloromethane. The resultant nanoparticles are then accumulated and purified through three rounds of washing with distilled water, each involving centrifugation at 15,000 rpm for 5 min. And then these nanoparticles are resuspended in deionized water for future use. To prepare dye-loaded nanoparticles, either a Dir solution (Invitrogen, Carlsbad, California, USA) or a rhodamine B (RDB) solution is added to the PLGA nanoparticle suspension, yielding Dir-loaded (PLGA^Dir^) or RDB-loaded nanoparticles (PLGA^RDB^), respectively. The nanoparticles containing Dia are evaluated for their physical properties. Sizes are measured utilizing DLS with a Mastersizer 3000 analyzer from Malvern Instruments Ltd. The encapsulation efficiency of Dia-loaded nanoparticles is 74%. The loading rate of diacetylene nanoparticles is 16%.

### 2.4 Release of dia from encapsulated nanoparticles

50 mg Dia (Sigma-Aldrich, St. Louis, USA) and 200 mg PLGA are prepared into nanoparticles following the aforementioned approach. Evenly disperse it into 84 equal segments and distribute these segments into four distinct groups: pH = 7, temperature (T) = 37 °C; pH = 7, T = 4 °C; pH = 5, T = 37 °C; pH = 5, T = 4 °C. Each set comprises 21 specimens, which are scheduled for examination spanning a period of 7 days. By evaluating the drug's encapsulation efficacy, the total quantity of Dia in each dose has been determined. We detected the Dia content by a spectrophotometer (DeNovix, USA) at a wavelength of 254 nm. Then following the steps in Fig. S1A to check the daily residual amount of the medication. Subtracting the daily residual amount from the total amount can obtain the daily release of Dia.

### 2.5 Culture of primary tendon cells and macrophages

Four-week-old SD rats are humanely sacrificed following anesthesia. The ankle joint region is scrupulously cleansed and disinfected with utmost precision. Subsequently, an incision is crafted to reveal the Achilles tendon, which is meticulously excised for subsequent examination. Following thorough rinsing in sterile PBS for over half a dozen iterations, the Achilles tendon tissue is then meticulously dissected into minute fragments using sterile scissors, and these pieces are then immersed in a 5 mg/mL Col-I solution. After 2 h, the tissue is digested into a flocculent material. The sample is filtered through a 70 μm strainer into a 50 mL tube and spun at 1000 g for 5 min. The supernatant, with non-adherent cells and debris, is discarded. The cell pellet is then constituted in a growth medium of 90% DMEM, 10% FBS, 100 mg/mL streptomycin, and 100 Units/mL penicillin. This cell suspension is then switched to culture dishes and enabled to grow in a controlled environment at 37 °C with 5% CO_2_ atmosphere.

Eight-week-old SD rats are subjected to fasting one day prior to the procedure and are subsequently euthanized under anesthesia. The rats are then swiftly wiped and disinfected before being moved to a sterile work surface. Utilizing sterilized scissors and forceps, the fur is inclined to reveal the abdominal muscles. Following a disinfection step with medical cotton swabs, approximately 15 mL of a 1640 medium is inoculated into the peritoneal cavity with a syringe. The abdominal area is gently massaged with a medical cotton swab for 3 min, and then left to rest for 5 min. Subsequently, a fresh pair of scissors is utilized to create a small incision in abdominal muscles. Sterile pipettes aspirate the peritoneal exudate and medium. After subjecting the cellular material to centrifugation at a velocity of 1000 rpm for a duration of 5 min, the cells are carefully rehydrated and subsequently placed into a culture dish to commence incubation. After 4 h, the medium was replaced with fresh 1640 medium.

### 2.6 Cell viability

The viability of rat Achilles tendon cells in response to various concentrations of Dia is assessed using the CCK-8 (Beyotime Biotechnology, Shanghai, China). The cells are seeded in a 96-well microplate and reached a confluence of 80% prior to Dia treatment. At the 24-h and 48-h pionts, the culture medium is aspirated, and replaced with 100 μL of new medium supplemented with 10 μL of CCK-8 solution. After another 2-h incubation, the samples' optical density is measured at 450 nm using a spectrophotometer.

### 2.7 TUNEL staining

The TUNEL staining technique, provided by Beyotime Biotechnology based in Shanghai, China, is employed to evaluate cellular apoptosis. Rat Achilles tendon cells are seeded in 96-well plates, treated with 30 μM Dia for 24 h, and exposed to 1 × 10^−4^ M H_2_O_2_ (Sigma-Aldrich, St. Louis, USA) for 4 h. The cells are subsequently permeabilized with proteinase K, and the TUNEL reaction mixture is implemented for a 60-min incubation period. Lastly, the cells are observed and recorded through a fluorescence microscope (Leica DMR 3000; Leica, Bensheim, Germany).

### 2.8 β-Galactosidase staining

To assess cell senescence, the β-galactosidase staining protocol (Beyotime Biotechnology, Shanghai, China) is employed. Rat Achilles tendon cells are seeded in 12-well plates at 5 × 10^5^ cells per well, treated with 30 μM Dia for 24 h, and then exposed to 1 × 10^−4^ M H_2_O_2_ for 4 h. After rinsing with PBS, the cells are fixed with β-galactosidase staining fixative for 15 min. After the fixative is removed, the cells underwent three PBS washes before the application of the staining working solution, which is left to incubate at 37°C for an extended period. The percentage of β-galactosidase-positive cells is calculated by counting 150–200 cells in six randomly chosen microscopic fields.

### 2.9 γ-H2AX staining

The γ-H2AX staining technique, provided by Beyotime Biotechnology based in Shanghai, China, is employed to evaluate cellular DNA damage. Rat Achilles tendon cells are seeded in 96-well plates, treated with 30 μM Dia for 24 h, and exposed to 1 × 10^−4^ M H_2_O_2_ for 4 h. The cells are blocked at room temperature for 15 min, and incubated overnight at 4 °C with the primary antibody against γ-H2AX. Then the cells are incubated with Cy3 goat anti-rat secondary antibody at room temperature for 45 min. The immunostained sperm were counterstained with DAPI and observed and recorded through a fluorescence microscope (Leica DMR 3000; Leica, Bensheim, Germany).

### 2.10 ROS level assay

ROS levels in tendon cells are discovered with the ROS Assay Kit (Beyotime Biotechnology, Shanghai, China), following instructions. Briefly, the cells are first treated with 30 μM Dia for 24 h, followed by a 4-h exposure to 1 × 10^−4^ M H_2_O_2_ in a serum-free DMEM. The fluorescent probe is then combined with the serum-free medium and adjusted to the appropriate working dilution. Following a 20-min incubation period, the cells are examined and their fluorescence is recorded using a fluorescence microscope.

### 2.11 Western blot assay of cell protein

Utilizing RIPA lysis buffer, which is supplemented with 1% protease inhibitor, across diverse rat Achilles tendon cell populations. Upon lysate collection, incorporate 1 × loading buffer and proceed to denature the proteins via boiling. Subsequently, store the proteins at a standard −20°C for preservation. Protein extracts are resolved by SDS polyacrylamide gel using a gradient gel with concentrations ranging from 6% to 12%. Following electrophoresis, proteins are transferred to a PVDF membrane preincubated with 5 % non-fat milk for 1.5 h. The membrane is incubated overnight at 4°C with Col I (1:1000, Proteintech, China), Bax (1:1000, Proteintech, China), SCX (1:1000, Proteintech, China), p16 (1:1000, Proteintech, China), p21 (1:1000, Zenbio, China) and p53 (1:1000, Cell signaling technology, USA). Then the PVDF membrane is washed, and incubated again overnight with IRDye800 secondary antibody (1:10000, Rockland Gilbertsville, CA, USA) at 4 °C. Protein bands are detected using the Odyssey system and quantified with Image J software, normalized to β-actin (1:10000, Proteintech, China).

### 2.12 Proteomic analysis of tendon cells

Rat Achilles tendon cells are seeded in 6-well plates, treated with 30 μM Dia for 24 h, and exposed to 1 × 10^−4^ M H_2_O_2_ for 4 h. Then the cell samples are collected and stored at −80 °C were thawed on ice and lysed using 8 M urea buffer containing 1% protease inhibitor, followed by sonication and centrifugation (15,000×*g*, 10 min, 4°C) to obtain clarified supernatants for BCA protein quantification, after which two parallel digestion approaches were implemented - the precipitation method involving TCA precipitation (20% final concentration), acetone washes, TEAB resuspension, and tryptic digestion (1:50, 37°C overnight) following reduction (5 mM DTT, 56°C, 30 min) and alkylation (15 mM IAA, 15 min dark), and the FASP method utilizing centrifugal filters for buffer exchange after reduction/alkylation with sequential tryptic digestions (1:50 overnight + 1:100 for 4 h) in HEPES buffer, with both methods yielding peptides that were acidified (10% TFA to pH 2–3), desalted using Strata X columns, quantified by Pierce assay, and vacuum-dried before TMT labeling in 100 mM HEPES buffer (1 h RT) with labeling efficiency verified by mass spectrometry prior to reaction quenching with hydroxylamine and high-pH reverse-phase HPLC fractionation (Agilent 300Extend C18 column, pH 9.0 mobile phases) employing a 54-min 6–32% acetonitrile gradient with fraction pooling into 16 samples for lyophilization, followed by LC-MS/MS analysis on either a DDA platform (Easy-nLC 1200 coupled to Q Exactive HF-X using 20 cm C18 column with 7–80% acetonitrile gradient over 40 min and TOP-20 HCD fragmentation at 15k resolution) or a DIA platform (Vanquish Neo-Astral system with 380–980 *m/z* MS1 scans at 240k resolution and 299 DIA windows at 2 *m/z* isolation), with resulting data processed through Maxquant (DDA) or DIA-NN (DIA) against UniProt databases using tryptic cleavage rules (max 2 missed cleavages), fixed carbamidomethylation, variable modifications (oxidation, acetylation), and 1% FDR thresholds for protein identification, followed by comprehensive bioinformatics analysis including GO term annotation (cellular components, molecular functions, biological processes) and KEGG pathway mapping for functional enrichment of DEPs that were further subjected to cluster analysis (Z-score transformed -log10 p-values, Euclidean distance metric) and protein-protein interaction network construction using STRING (confidence score >0.7) visualized via visNetwork to elucidate biological pathways and molecular relationships within the proteomic dataset. The proteomic analysis was examined by the Cosmos Wisdom (Zhejiang, China).

### 2.13 Immunofluorescence for macrophages

Macrophages from various experimental groups are originally rinsed twice using PBS and then fixed using a 4 % PFA solution. Following this, non-specific binding is blocked with 1 % BSA. The samples are incubated at 4 °C with anti-CD206, anti-CD86, and anti-CD68 (Proteintech, China) at a 1:200 dilution on the whole night. After PBS rinsing, the specimens are subsequentlycon exposed to secondary antibodies, diluted to the same extent, and allowed to incubate for a duration of 1 h at room temperature. The cellular nuclei are meticulously counterstained with DAPI for a duration of 5 min, following which each slide is meticulously captured under the scrutiny of a fluorescence microscope.

### 2.14 Flow cytometry

Macrophages are gently dissociated using Trypsin-EDTA, followed by quelling with 1640 medium. Upon centrifugation, the cells are reconstituted in 200 μL of PBS. The methodology for macrophage identification has been delineated in our prior publications [27]. In this study, F4/80 is utilized to identify macrophages, while iNOS is accustomed to identify M1 macrophages, and CD163 was employed to recognize M2 macrophages. Then, anti-rat iNOS (1 μL/100 μL 1 × 10^7^ cell suspension) (Invitrogen, Carlsbad, CA, USA) and anti-rat CD163 (1 μL/100 μL 1 × 10^7^ cell suspension) (BioLegend®, San Diego, CA, US) are added in two separate cell suspensions and incubate in the dark at room temperature for 15 min. There is no staining in the care. Finally, stained and unstained cells are tested on a flow cytometer (Attune NxT, Invitrogen) and the percentage of cells is calculated. The assessment of F4/80 and iNOS is conducted via the PE channel, whereas the examination of CD163 is carried out through the FITC channel.

### 2.15 RNA-sequencing

Total RNA preparation and subsequent RNA-seq library construction are executed with the APExBIO Technology LLC (Shanghai, China) service. Briefly, total RNA is isolated using a commercial kit (Tiangen Biotech, DP424), and RNA libraries are established via an RNA cleaning and concentration kit (APExBIO Technology LLC, K1159) after quality inspection and purity testing. The integrity of the RNA samples is confirmed through the utilization of an Agilent 2100 Bioanalyzer (Agilent Technologies, Santa Clara, CA). The selected libraries are processed through Illumina NovaSeq 6000 for paired-end sequencing, ensuring the acquisition of 150 bp read length sequences, in accordance with the determined effective concentration and desired data yield. Then, the Transcripts Per Million (TPM) data format of gene expression and the Limma R package are utilized to perform background correction, normalization, log2 transformation, and differential analysis. The criteria for screening DEGs are set as an adjusted p-value (adj.p) below 0.05 and log2FC greater than 1. The clusterProfiler R package was utilized to perform GO and KEGG enrichment analysis. Subsequently, Gene Set Enrichment Analysis (GSEA) was conducted to assess the genes' distribution pattern within a specified gene set, as aligned with the gene list ordered by the level of association with the phenotype, thus elucidating their role in shaping the phenotype. GSEA enrichment analysis is utilized to identify the signaling pathways that are significantly activated or inhibited in the LPS + Dia group.

### 2.16 ELISA assay

Assays are meticulously carried out to assess oxidative stress indicators, the efficacy of antioxidant defenses, and the concentration of inflammatory cytokines, all through the precision of ELISA methodologies. Subsequent to collection of cell culture supernatants and tissue homogenates, the levels of ROS/RNS are meticulously gauged utilizing the OxiSelect ROS/RNS assay kit (Cell Biolabs, Inc, California, USA). Additionally, the levels of MDA and SOD are evaluated utilizing ELISA assay kits (Beyotime Biotechnology, Shanghai, China), adhering to the standard operating procedures. The quantification of cytokines pertaining to inflammation is meticulously conducted using ELISA kits (Jiangsu Jingmei Biotechnology Co., Ltd, Yancheng, China), in accordance with the manufacturer's guidelines.

### 2.17 Production and characterization of DMN patches

Low molecular weight hyaluronic acid (Bloomage Biotechology Co., Ltd. Jinan, China) dissolves in various concentrations of Dia-encapsulated nanoparticles solution. Centrifuging at 4000 g for 20 min to mix thoroughly. Subsequently, the blend is meticulously transferred into a polydimethylsiloxane (PDMS) matrix, followed by securing it with a cover. The assembly is then subjected to centrifugal force at 4000g for a duration of 20 min, after which the cover is meticulously removed. Upon complete desiccation, the microneedles are primed for application onto the surface of genuine leather. The morphologies of DMNs are characterized using a mobile phone camera, SEM and an ultra-depth 3D microscope (Leica, Germany). DMN/PLGA^RDB^ is observed through that microscope made in Germany. The texture analyzer is employed to quantify the hardness, adhesive force, resilience, cohesive properties, and spring-like characteristics of MNs. (FTC TMS-PRO, USA).

### 2.18 Animal model

The animal experiment protocols are assisted by the IACUC at Nantong University School of Medicine (code S20240420-191). Sixty eight-week-old Sprague-Dawley rats were employed to establish a rat model that replicates the condition of Achilles tendon enthesis injury. The creatures are methodically distributed into five distinct experimental groups: a Control group serving as the baseline; a group subjected to Surgical Repair; a NC group which receives weekly administrations of DMN/PLGA subsequent to surgical intervention; a Low Dose group that is administered weekly low-dose DMN/PLGA@Dia following their surgery; a Medium Dose group treated with weekly medium-dose DMN/PLGA@Dia in the aftermath of surgery; and a High Dose group that receives weekly high-dose DMN/PLGA@Dia post-operatively. Each group consists of 12 rats. The experimental schedule dictates that six rats from each group are sacrificed at the 4-week mark, while the remainder are humanely euthanized at the 8-week mark. In the Achilles tendon-to-bone insertion injury model, the study encompasses both ankle joints of each rat. An L-shaped incision is meticulously made on the lateral aspect of the ankle joint, through which the skin is carefully opened. Utilize hooks to meticulously dissect the soft tissue, gradually revealing the calcaneus and the layers of the Achilles tendon. The surgical technique entails a partial incision of the Achilles tendon, situated roughly 4 mm above its bony attachment. Two minuscule intramedullary tunnels, each with a diameter of 0.5 mm, are created within the calcaneus by employing a compact electric drill. The reattachment of the Achilles tendon to its original anatomical position is achieved with the utilization of 5-0 prolene sutures (manufactured by Ethicon, located in Somerville, New Jersey, USA), employing an enhanced Mason Allen suturing technique. Seal the muscle incision meticulously with 5-0 polypropylene sutures. Secure the closure with a fine 4-0 black silk thread. Administer 80,000 units of penicillin intravenously for a consecutive three-day period postoperatively. Following one week of recovery, once the ankle edema has resolved, the rats will undergo DMN/PLGA@Dia therapy. Assess the histological alterations surrounding the repair insertion by employing the Modified Histomorphometric Scoring System and the Modified Bonar Score [28,29].

### 2.19 CatWalk

To test the functional recovery of the rats, CatWalk tests are performed at 4 and 8 weeks. The CatWalk system features a clear, illuminated walkway, bathed in the glow of a white fluorescent light source, which is designed for internal reflection. When a subject steps onto the glass surface, the light is deflected downward, where it is subsequently captured by a camera. The imagery thus obtained is digitized and meticulously analyzed via the sophisticated CatWalk analysis software. The system is adept at evaluating a variety of static and dynamic gait attributes. Metrics like the average claw pressure, duration of stance, and period of swing serve as proxies for detecting mechanical hyperalgesia and neuropathic pain [30,31]. In this study, meticulous examination of distinct paw parameters is conducted, focusing on the stance durations of the right and left paws (RH and LH, respectively), the velocities of the paw swings for both the RH and LH, and the stride lengths exhibited by each paw. The analysis of the recorded video footage is carried out using the CatWalk software, version 9.0 for Windows, with the evaluators remaining unaware of the group assignments in the experiment.

### 2.20 *In vivo* evaluation of the release of nanoparticles after microneedle insertion

To evaluate the local release of nanoparticles inside microneedles after insertion into the skin, DMN/PLGA^Dir^ is put on the surface of the normal rat ankle joint skin. Following the initial procedures, in vivo imaging studies are administered using the Tanon ABL X6. Imaging sessions are scheduled on 1d, 2d, 3d, 4d, 5d, 6d, and 7d. Specifically, on 3d and 7d, the rats' ankle joints are excised and the overlying skin is carefully removed to facilitate in vitro imaging. Additionally, the vital organs of the rats were meticulously gathered for subsequent in vivo imaging analysis.

### 2.21 Biomechanical testing

With meticulous care, separate the sample from the surrounding tissues and remove it completely. Utilize an Instron universal testing machine to evaluate the tensile strength of the recovering tissue (Fig. S2). Fasten the distal and proximal segments of the Achilles tendon securely within the lower and upper clamps, respectively, ensuring the repair zone is centrally located between the two clamps. Apply tension to the upper grip, securing one end of the tendon, at a consistent pace of 25 mm per minute, and terminate the test when the tendon's osseous junction reaches failure. The subsequent load-displacement graph, generated by Series IX testing software (also provided by Instron), demonstrates a marked decline, indicating the precise moment of failure at the repair site.

### 2.22 Western blot assay of tissue protein

The area of the Achilles tendon situated near the calcaneus is meticulously treated with RIPA lysis buffer, supplemented with a 1% protease inhibitor. Following the collection of the lysate, 1 × loading buffer is added prior to denaturing the proteins through boiling. Proteins are stored normally at −20°C. Protein isolates are effectively separated using a gradient SDS polyacrylamide gel ranging from 6% to 12%. Subsequently, the proteins are transferred onto a PVDF membrane, followed by a pre-incubation with 5% non-fat milk for a duration of 1.5 h. Thereafter, the membrane is incubated with antibodies specific to Collagen I and Bax (both 1:1000, Proteintech, China) whole night at 4°C. The next day, the membrane is rinsed and incubated with IRDye800 (1:10000, Rockland Gilbertsville, CA, USA) secondary antibody conjugated whole night at 4°C. The membrane is detected by the Odyssey system, and their density is quantified with Image J software, normalized to β-actin (1:10000, Proteintech, China).

### 2.23 Masson's trichrome, safranin O staining and HE staining

Procure ankle joint specimens from various rat groups, and fix them with 4% paraformaldehyde at ambient temperature for a duration of 24 h to ensure stabilization. Subsequently, execute decalcification meticulously. Proceed to trim 5 μm thick sections utilizing a paraffin microtome, followed by rehydration. Conclude by conducting Masson trichrome, Safranin O, and HE staining procedures (Servicecbio Technology Co., Ltd, Wuhan, China). Following the processing of stained tissue samples, an analysis is conducted to assess inflammation and the organization of collagen fibers, focusing on their distribution and accumulation within the tissues. Tissue sections from rat hearts, livers, spleens, lungs, and kidneys in different experimental groups are paraffin-embedded for histological analysis. These sections are subjected to HE staining to ascertain the tissue reaction, which in turn facilitates the assessment of the DMN/PLGA@Dia treatment's biocompatibility.

### 2.24 Sirius red staining

As previously indicated, 5 μm thick tissue sections were meticulously prepared with a paraffin microtome and subsequently rehydrated to facilitate immunohistochemical examination. Following rehydration, Sirius Red staining was executed in conformity with established protocols (Solarbio, Beijing, China). By evaluating collagen birefringence and cartilage formation under a polarized light microscope (Leica), differences in collagen and cartilage deposition at the enthesis can be detected. The rotation of the microscope stage is meticulously set to a consistent angle for the acquisition of all images, as any variation in stage rotation has the potential to alter the hue of the collagen fibers. Notably, the finer and disrupted fibers exhibit a greenish tint, indicative of Col III or fibrochondrocytes, whereas the more robust fibers are characterized by an orange-yellow staining, representing Col I.

### 2.25 Immunohistochemical staining

As previously outlined, tissue sections of 5 μm are prepared via a paraffin microtome and subjected to rehydration in preparation for immunohistochemical examination. After blocking, the sections are incubated with Col I and Col III (both 1:200, Proteintech, China) at 4°C whole night. Subsequently, they were incubated with a secondary antibody for a duration of 2 h at a temperature of 37 °C. Following this, the sections were meticulously stained using 3,3′-DAB (Servicecbio Technology Co., Ltd, Wuhan, China) to visualize the immunoreactivity, followed by hematoxylin for nuclear staining. Once the staining process is complete, the slides are mounted, covered through a microscope (Leica DMR 3000; Leica, Bensheim, Germany).

### 2.26 Immunofluorescence staining

Following rehydration, tissue sections are permeabilized with 0.3 % Triton X-100 for 1.5 h and stained with CD68 and CD206 primary antibodies (1:200, Proteintech, China) whole night at 4°C. Slides are then treated with the secondary antibody at 37°C for another 2 h. After washing, they are stained with DAPI as well as found through a fluorescence microscope (Leica DMR 3000; Leica, Bensheim, Germany).

### 2.27 Statistical analysis

Results are expressed as mean ± SD. One-way ANOVA is utilized to assess variations among three or more groups with unequal variances. In instances where significance was detected, Fisher's least significant difference test was employed for subsequent pairwise comparisons. Statistical significance was defined as the P < 0.05 level.

## 3 Results and discussion

### 3.1 Characterization of DMN/PLGA@Dia and the evaluation of the release of nanoparticles after microneedle insertion

**Fig. 1:**
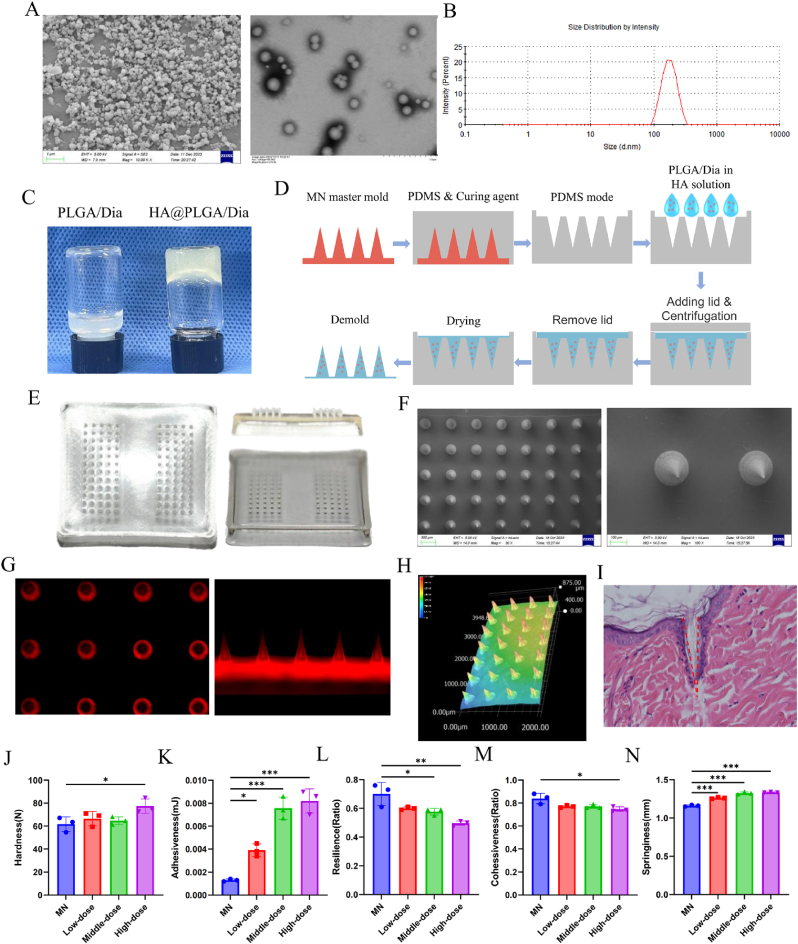
**Characterization of DMN/PLGA@Dia.** (A) Scanning electron microscopy (left) and transmission electron microscopy (right) images of drug-encapsulated nanoparticles. (B) Nanoparticle tracking analysis of drug-encapsulated nanoparticles. (C) Liquidity observation after addition low molecular weight hyaluronic acid (200 mg/mL). (D) The preparation process of DMN/PLGA@Dia. (E) General appearance of DMN/PLGA@Dia. (F) Scanning electron microscopy images of DMN/PLGA@Dia. (G) Typical fluorescence images of DMN/PLGA^RDB^ in cross section and longitudinal section. (H) Typical ultra-depth microscopy images of DMN/PLGA@Dia. (I) H&E staining image of the skin after microneedle insertion. (J–N) The hardness (J), adhesiveness (K), resilience (L), cohesiveness (M) and springiness (N) of DMN/PLGA with different concentrations of Dia (n = 3). ∗P < 0.05; ∗∗P < 0.01; ∗∗∗P < 0.001.

As documented in our prior investigations, the resultant drug-encapsulated nanoparticles exhibit a spherical morphology, with an average particle diameter of 181.8 ± 48.51 nm (Fig. 1A and B) [24]. Upon thorough blending with low molecular weight hyaluronic acid at a concentration of 200 mg/mL, the resultant mixture assumes a gelatinous consistency (Fig. 1C). Then, adhering to the procedure illustrated in Fig. 1D, fabricate DMN/PLGA@Dia. Fig. 1E exhibits the general appearance of microneedle. Each micro-spike features a configuration of 2 × 5 × 12 spike tips, and is split into two distinct arrays. Upontrimming the borders, it can be evenly allocated for application on both ankle joints of a rat, with a fitting dimension. The needles have conical shapes, as shown by ultra-depth microscope images (Fig. 1H) and SEM images (Fig. 1F). DMN/PLGA^RD^^B^ shows that the nanoparticles can be uniformly distributed in the microneedles (Fig. 1G). H&E staining displays that the channels can be formed in the skin by DMN/PLGA@Dia patch (Fig. 1I). The texture analyzer findings revealed that the DMN/PLGA@Dia exhibited a partial enhancement in hardness when contrasted with the untreated microneedle (Fig. 1J). The enhanced strength of microneedles is attributed to the integration of nanoparticles, aligning with our findings that corroborate earlier research conclusions [24]. The adhesiveness and springiness are significantly increased (Fig. 1K and N). The resilience and cohesiveness are partially decreased (Fig. 1L and M).

### 3.2 Dia alleviated oxidative stress and restored the specific differentiation potential of tendon cells in vitro

Firstly, the oxidative stress following tendon-bone insertion injury is evaluated. The GSE103266 dataset, consisting of 4 human control samples and 12 rotator cuff injury samples, is selected. Using the Limma package, a total of 233 down-regulated and 536 up-regulated DEGs are identified. (Fig. S3A and S3B). GO functional enrichment analysis shows that these DEGs are significantly associated with immune activation and oxidative stress signaling pathways (Fig. S3C). PPI protein network analysis reveals the crosstalk at the protein level of these DEGs (Fig. S3D). KEGG analysis is used to further elucidate the functions of these genes. The results of the KEGG enrichment analysis shows that oxidative stress pathways are highly enriched in the rotator cuff injury group, indicating that cellular oxidative stress may be involved in the repair process of acute rotator cuff injury (Fig. S3E). In the expression matrix, the mRNA expression level of IL-1β is separately analyzed. The findings reveal a marked elevation in the mRNA expression level of IL-1β subsequent to rotator cuff injury (Fig. S3F). To ascertain the resemblance between the Achilles tendon-bone insertion and the rotator cuff insertion, particularly in relation to the sustained oxidative stress that follows injury. The supernatant of Achilles tendon-bone insertion tissues, including those from uninjured tissues and tissues injured at 2W, 4W, and 8W, is extracted, and the content of ROS/RNS, MDA, IL-1β, and SOD is then detected. The findings indicate that following injury to the Achilles tendon-bone insertion, there is a persistent elevation in the levels of ROS/RNS, MDA, and IL-1β, while there is a noted reduction in the antioxidant stress enzyme, SOD (Fig. S3G).

**Fig. 2:**
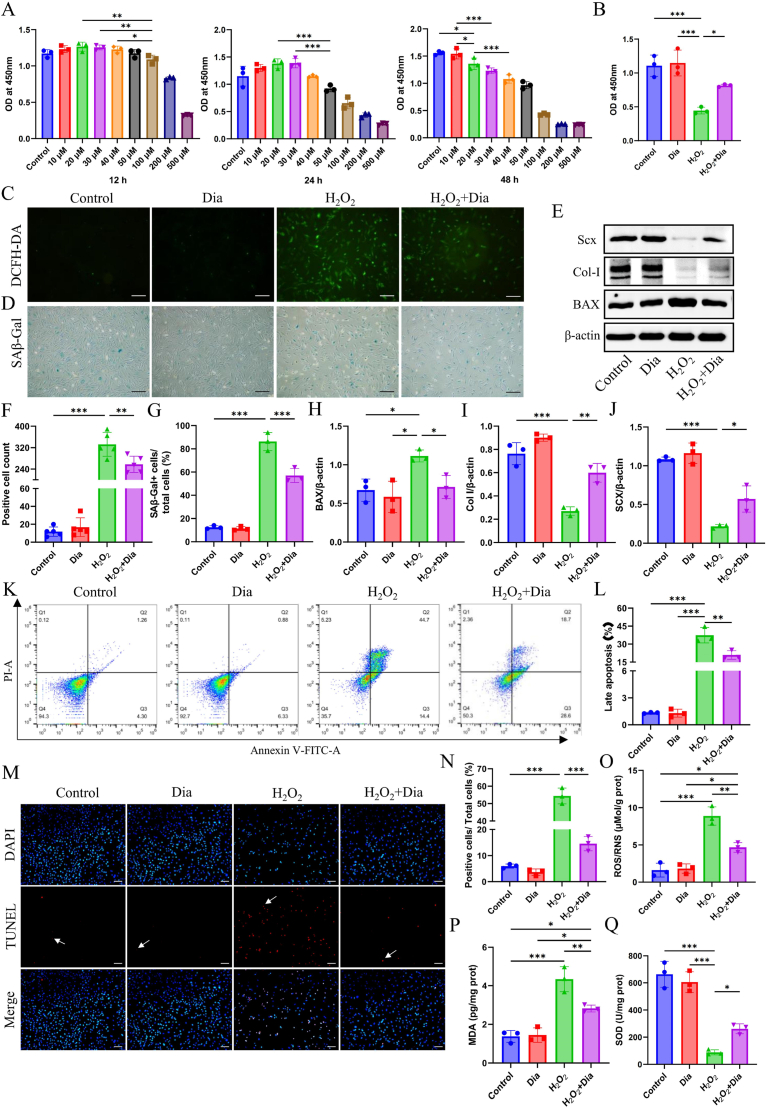
**Dia alleviated H_2_O_2_-induced oxidative stress and alleviated the inhibition of tendon-specific marker expression in tendon cells under oxidative stress in vitro.** (A) Cell viability of tendon cells exposed to different Dia concentrations at 12 h, 24 h and 48 h (n = 3). (B) Cell viability of tendon cells exposed to H_2_O_2_ and/or Dia (n = 3). (C) Representative images of intracellular ROS detection in tendon cells using DCFH-DA. Scale bar = 100 μm. (D) SA*β*-gal staining of Dia-treated tendon cells subjected to H_2_O_2_ stimulation. Blue cells are senescent. Scale bar = 100 μm. (E) Expression of SCX, Col-I and Bax in Dia-treated tendon cells subjected to H_2_O_2_ stimulation. (F) Quantitative analysis of the number of ROS-positive cells in C (n = 5). (G) Semi-quantitative analysis of the percentage of positive cells of D (n = 3). (H–J) Representative Western blot assay of SCX, Col-I and Bax in E (n = 3). (K) Annexin/PI-labeled flow cytometric analysis of apoptotic tendon cells under oxidative stress environment. (L) The proportion of late apoptosis in K (n = 3). (M) Immunofluorescence staining of TUNEL of apoptotic tendon cells under oxidative stress environment. The arrow points to positive cells. Scale bar = 100 μm. (N) Semi-quantitative analysis of the percentage of positive cells from M (n = 3). (O–Q) The content of ROS/RNS, MDA and SOD of Dia-treated tendon cells subjected to H_2_O_2_ stimulation measured by ELISA (n = 3). ∗P < 0.05; ∗∗P < 0.01; ∗∗∗P < 0.001. (For interpretation of the references to colour in this figure legend, the reader is referred to the Web version of this article.)

Then, the cell viability of tendon cells is measured at different concentrations of Dia at 12, 24, and 48 h. The findings indicate that tendon cells exhibited optimal viability when incubated with a concentration of 30 μM Dia for a duration of 24 h (Fig. 2A). Therefore, in the subsequent experiments, Dia at a concentration of 30 μM will be used to pre-stimulate tendon cells for 24 h in all Dia-treated groups. In this experiment, tendon cells are stimulated with 1 × 10^−4^ M hydrogen peroxide (H_2_O_2_) for 4 h to simulate an oxidative stress microenvironment in vitro. Upon exposure to H_2_O_2_, the viability of standard tendon cells witnesses a marked decline; however, pre-treatment with Dia is found to mitigate this adverse impact. (Fig. 2B). Then the total ROS levels are detected by DCFH-DA (Fig. 2C). The findings indicate that the proportion of DCFH-DA fluorescence-positive cells in the H_2_O_2_ group is markedly elevated compared to the Control group. Moreover, the H_2_O_2_+Dia group exhibited a considerable reduction in the relative number of DCFH-DA fluorescence-positive cells in relation to the H_2_O_2_ group alone (Fig. 2F). Besides, the content of ROS/RNS, MDA, and SOD is also measured by ELISA in Dia-treated tendon cells subjected to H_2_O_2_ stimulation (Fig. 2O-Q). The findings indicate that the ROS/RNS and MDA concentrations are markedly elevated, whereas SOD level witnesses a significant decline in the H_2_O_2_ group relative to the Control and Dia groups. Treatment with Dia ameliorates this alteration.

Drawing on prior studies, it has been observed that H_2_O_2_ can mimic an oxidative stress milieu, thereby suppressing the proliferation and differentiation of tendon cells [8]. Here, Dia with ROS scavenging ability is used to investigate whether it can reduce the inhibitory effect of H_2_O_2_ on the tendon differentiation potential of tendon cells. Col-I is the major component of the extracellular matrix synthesized by tendon cells in normal tendons. Furthermore, SCX serves as a pivotal transcription factor in the process of tendon development, playing a crucial role in the differentiation of tendon lineage cells [32]. The expression levels of Col-I and SCX in different groups of tendon cells are detected by Western blot (Fig. 2E). The findings indicate that the levels of Col-I and SCX expression are markedly reduced in the H_2_O_2_ group, in contrast to the Control and Dia groups. Tendon cells pretreate with Dia managed to preserve a degree of their differentiation potential even when subjected to an oxidative stress environment. (Fig. 2I and J).

Severe oxidative stress can lead to apoptosis of tendon cells [33]. In our study, apoptotic tendon cells are analyzed using Western blot, TUNEL staining, and Annexin V/PI-labeled flow cytometry. The western blot analysis reveals a marked elevation in Bax expression within the H_2_O_2_ group, as opposed to the Control and Dia groups. Conversely, the H_2_O_2_+Dia group exhibits a substantial reduction in Bax expression when compared to the H_2_O_2_ group alone (Fig. 2E and H). The rate of late apoptosis positive tendon cells decreases from 37.4 ± 5.22% in H_2_O_2_ group (Fig. 2K and L) to 21 ± 2.93% in H_2_O_2_+Dia group. The rate of TUNEL-positive cells is also significantly reduced (Fig. 2M and N).Furthermore, our results of γ-H2A.X staining showed that Dia can alleviate DNA damage in tendon cells caused by H_2_O_2_ (Fig. S4A and S4B). Senescence-associated beta-galactosidase (*β*-gal) staining displays that Dia inhibits the senescence of tendon cells (Fig. 2D and G). The WB results showed that compared to the H_2_O_2_ group, Dia significantly reduced the expression of p16. However, there was no significant difference in the expression of p21 and p53 among several groups (Fig. S4C and S4D).In order to further investigate the mechanism by which Dia reduces oxidative stress in tendon cells, we conducted proteomic analysis on tendon cells in each group. PCA analysis shows that these three groups have significantly different proteomic differences (Fig. S5A). Further differential analysis showed that the H_2_O_2_ group had a total of 385 upregulated proteins and 627 downregulated proteins compared to the Control group (Fig. S5B). The H_2_O_2_+Dia group had a total of 116 upregulated proteins and 162 downregulated proteins compared to the H_2_O_2_ group (Fig. S5C). Subsequently, we intersected the proteins with lower expression in group H_2_O_2_ compared to group Control, as well as the proteins with higher expression in group H_2_O_2_+Dia compared to group H_2_O_2_, and created an intersection protein heatmap (Fig. S5D). The results showed that Lipoic Acid Synthetase (Lias) and Solute Carrier Family 23 Member 2 (Slc23a2) proteins were lowly expressed in the H_2_O_2_ group, while they were relatively highly expressed in the Control and H_2_O_2_+Dia groups. Lias is a key enzyme involved in the synthesis of lipoic acid, which is a potent antioxidant that can neutralize free radicals and protect cells from oxidative damage. Studies have shown that Lias plays a crucial role in regulating the redox balance within cells, and its deficiency may increase cellular sensitivity to oxidative stress [34,35]. Slc23a2 is a member of the vitamin C transporter family, responsible for transporting vitamin C into cells. Vitamin C is an important antioxidant that effectively eliminates free radicals and protects cells from oxidative stress damage. The expression and function of Slc23a2 may influence the cell's uptake of vitamin C, thereby regulating its antioxidant capacity [36,37]. The GO and KEGG functional enrichment analysis of intersecting proteins showed that these proteins are mainly closely related to oxidative stress-related pathways (Fig. S5E and S5F). PPI analysis further revealed significant connections between these intersecting proteins (Fig. S5G). These results suggest that Dia may alleviate oxidative stress by upregulating the expression levels of Lias and Slc23a2 proteins. However, more experiments are needed for verification.

### 3.3 Dia adjusted macrophage M1/M2 polarization *in vitro*

**Fig. 3:**
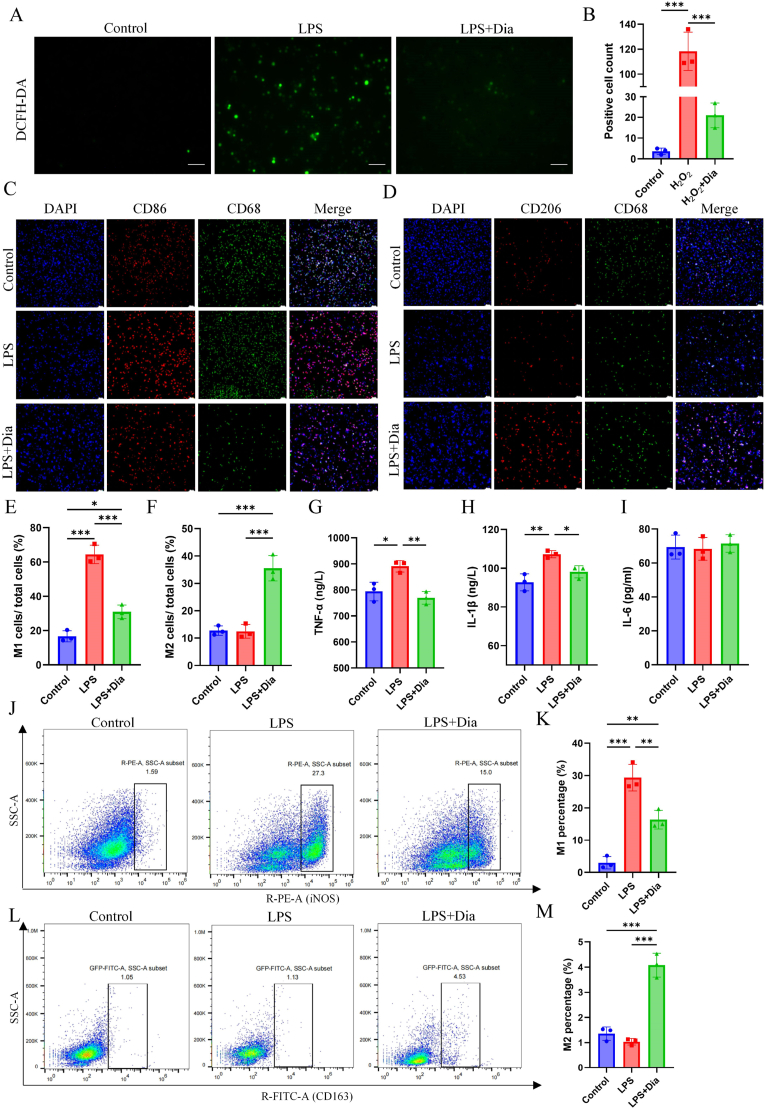
**Macrophages polarization-regulating effects of Dia under oxidative stress in vitro.** (A) Representative images of intracellular ROS detection in primary macrophages using DCFH-DA. Scale bar = 50 μm. (B) Quantitative analysis of the ROS-positive cell counts in A (n = 3). (C) Representative immunofluorescent images of total and M1 polarised macrophages. Scale bar = 50 μm. (D) Representative immunofluorescent images of total and M2 polarised macrophages. Scale bar = 50 μm. (E) Semiquantitative analysis of the percentage of M1 cells from C (n = 3). (F) Semiquantitative analysis of the percentage of M2 cells from D (n = 3). (G–I) The content of TNF-α, IL-1β and IL-6 in Dia-treated macrophages under LPS stimulation measured by ELISA (n = 3). (J) Flow cytometric analysis of M1 polarised macrophages. (K) Data analysis of J (n = 3). (L) Flow cytometric analysis of M2 polarised macrophages. (M) Data analysis of L (n = 3). ∗P < 0.05; ∗∗P < 0.01; ∗∗∗P < 0.001.

The flow cytometric analysis reveals that the isolated cells predominantly express F4/80 (at a rate of 86.6%), suggesting that the majority of the harvested cells are macrophages in nature (Fig. S6). Lipopolysaccharide (LPS) has the capability to activate M1 macrophage polarization in a manner that is contingent upon ROS, serving as a well-established in vitro model for pro-inflammatory responses. Here, this model is used to evaluate the ROS clearance and macrophage polarization regulation effects of Dia on primary macrophages. DCFH-DA is utilized to detect the total ROS level, and LPS induction significantly triggers the accumulation of ROS in macrophages. Dia has been shown to effectively inhibit LPS induced ROS production and ROS clearance (Fig. 3A and B). Investigating macrophage polarization via immunofluorescence labeling of CD86, indicative of M1 macrophages, and CD206, a marker for M2 macrophages. Following stimulation with LPS, there is a marked intensification in the fluorescence of CD86, whereas the CD206 fluorescence significantly diminishes. However, Dia treatment significantly increases the expression of CD206 (Fig. 3C-F). Furthermore, the ELISA findings indicate that, in contrast to the LPS group, the administration of Dia markedly diminishes the concentrations of the inflammatory cytokines TNF-α and IL-1β (Fig. 3G-H). Furthermore, the findings from flow cytometric analysis reveals that the expression levels of iNOS, a hallmark marker of M1 macrophages, are significantly reduced in the LPS + Dia group when contrasted with the LPS group. Concurrently, there is a notable enhancement in the expression of CD163, an indicative marker for M2 macrophages, in the LPS + Dia group in comparison to the LPS group (Fig. 3J-M). Those data all indicate that Dia can regulate macrophage polarization.

To further investigate the mechanism by which DMN@PLGA@Dia treatment affects macrophage polarization, RNA is extracted from macrophages treated with Control, LPS, and LPS + Dia for RNA sequencing. Principal component analysis (PCA) demonstrates distinct gene expression profiles among the three groups (Fig. S7A). Heatmap and volcano plot analyses have pinpointed a collection of genes with altered expression in the LPS + Dia group, when contrasted with the LPS and Control groups. In particular, the LPS + Dia group exhibits upregulation of 1455 genes and downregulation of 1699 genes in comparison to the Control group. In contrast to the LPS group, the LPS + Dia group exhibits an increase in the expression of 213 genes, while 103 genes show a decrease in expression (Fig. S7B–E). Venn diagram analysis has identified 86 genes that are commonly upregulated and 102 genes that are commonly downregulated in the LPS + Dia group as DEGs (Fig. S7F). Utilizing the clusterProfiler R package, a thorough analysis is conducted on the DEGs to perform GO and KEGG enrichment analysis. Findings from the GO enrichment reveals that the alterations in gene expression caused by LPS stimulation are predominantly linked to acute inflammatory responses, cytokine secretion pathways, the ERK signaling pathway, and the differentiation of macrophages (Fig. S7G). KEGG enrichment analysis showed that the alterations in gene expression caused by LPS stimulation are predominantly linked to NF-κB signaling pathway, Pl3K-Akt signaling pathway, Toll-like receptor signaling pathway et al. (Fig. S7H) GSEA further reveals that the acute inflammation pathway, NF-κB signaling cascade, and ERK signaling are significantly suppressed in the LPS + Dia group, while the TGF-β signaling pathway is notably activated (Fig. S7I–L). In congruence with the molecular underpinnings governing the regulation of macrophage M1/M2 polarization by Dia, the scientific literature has documented several studies. For instance, Zhang and colleagues have demonstrated that nanofibers derived from rhein, an active metabolite of Dia, are capable of stimulating the PI3K/AKT/mTOR signaling cascade, thereby diminishing oxidative stress. Additionally, these nanofibers suppress the NF-κB and STAT3 signaling pathways, exerting influence over inflammation and the polarization of macrophages [38]. Zheng et al. also found that rhein inhibited neuroinflammation through the inhibition of the NF-*κ*B signaling pathway [39]. Geng et al. discovered that WDR74 could facilitate TGF-β/Smad pathway activation to promote M2 macrophage polarization [40]. Therefore, it is hypothesized that the NF-κB signaling cascade, ERK pathway, and TGF-β signaling may play key roles in the Dia-induced M2 polarization of macrophages. Overall, these results provide a theoretical basis for Dia to regulate macrophage polarization.

### 3.4 The release of dia nanoparticles by DMN/PLGA@Dia

In vitro release studies have demonstrated that Dia nanoparticles are capable of releasing approximately 64.686 ± 0.066% of their content over a period of 7 days, when maintained at a pH of 7 and a temperature of 37°C. It is observed that a reduction in pH results in a modest increase in the release quantity, whereas a decline in temperature corresponds to a slight reduction in the amount released (Fig. S1B–D)

**Fig. 4:**
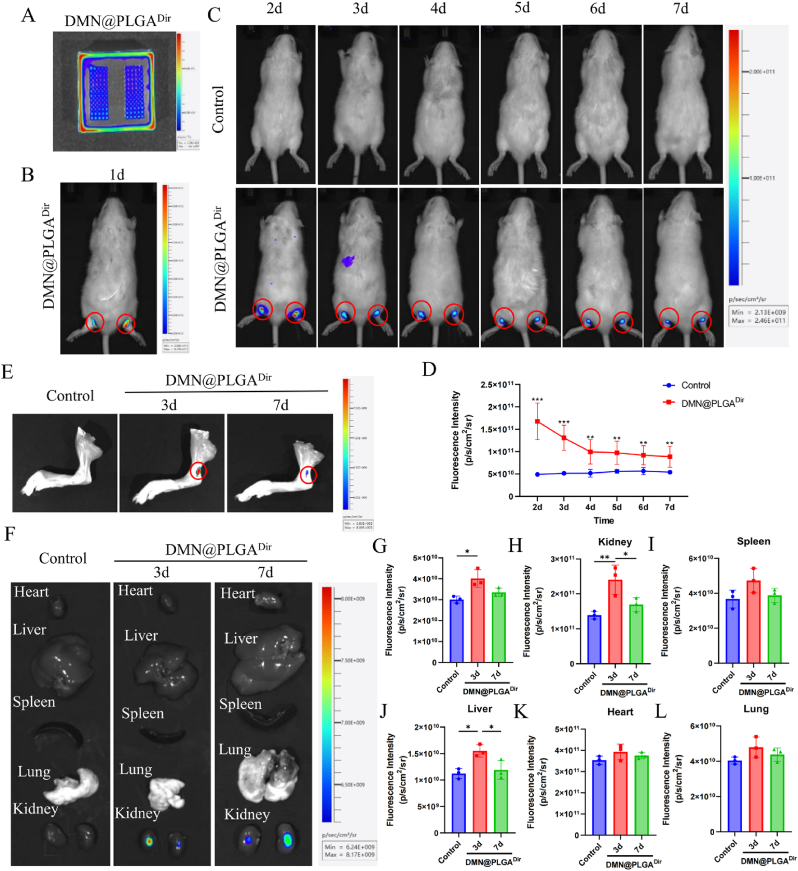
**Evaluation of nanoparticle release after DMN/PLGA@Dia insertion.** (A) Typical images of DMN/PLGA^Dir^. (B) *In vivo* images of rats 24 h after microneedle insertion. (C) Distribution and metabolism of PLGA^Dir^ in the body of rats after microneedle detachment within 7 days. (D) Data analysis of fluorescence intensity in C (n = 3). (E) *In vitro* images of PLGA^Dir^ in the ankle joint on days 3 and 7. (F) *In vitro* images of PLGA^Dir^ in the heart, liver, spleen, lung and kidney on days 3 and 7. (G) Data analysis of fluorescence intensity in the ankle joint (n = 3). (H) Data analysis of fluorescence intensity in kidney (n = 3). (I) Data analysis of fluorescence intensity in spleen (n = 3). (J) Data analysis of fluorescence intensity in liver (n = 3). (K) Data analysis of fluorescence intensity in heart (n = 3). (L) Data analysis of fluorescence intensity in lung (n = 3). ∗P < 0.05; ∗∗P < 0.01; ∗∗∗P < 0.001.

Then, to confirm that nanoparticles can be released from microneedles, we reconstructes DMN/PLGA^Dir^ (Fig. 4A). Tendon-bone injury is accordingly founded by a novel rat trans-calcaneal suture model. After reducing the swelling in the ankle joint, we placed DMN/PLGA^Dir^ on the surface of the rat ankle joint skin and performed in vivo imaging on 1d, 2d, 3d, 4d, 5d, 6d and 7d (Fig. 4B and C). To prevent the microneedles from being peeled off during rat activity, thin film patches and medical tape are used for reinforcement. However, despite these measures, most of the mice sheds off the microneedles within 48 h. After the microneedles are removed, fluorescence signals can still be detected at the ankle joint, indicating successful release of the nanoparticles within the microneedles. This is also one of the advantages of DMNs [21,41,42]. The quantitative analysis indicates that the fluorescence signal at the rat's ankle joint persisted for a duration of 7 days, exhibiting a continual decline over time (Fig. 4D). On 3d and 7d, the rat's organs are excised for subsequent in vitro fluorescence imaging. Post excision of the dermal layer, fluorescence remains discernible at the ankle joint (Fig. 4E). After 7 days, there is no significant difference in fluorescence intensity among the DMN/PLGA^Dir^ group and the Control group (Fig. 4G). The findings imply that the nanoparticles are capable of delivering medication to the subcutaneous region in a continuous and prolonged fashion. Additionally, the fluorescence quantitative assessment of the rats' hearts, livers, spleens, lungs, and kidneys has revealed that the primary metabolic sites for these nanoparticles are the liver and kidneys (Fig. 4F and 4H-4L).

### 3.5 DMN/PLGA@Dia reduced oxidative damage and promoted the healing of tendon-bone insertion tissues in a rat model of achilles tendon insertion point rupture

**Fig. 5:**
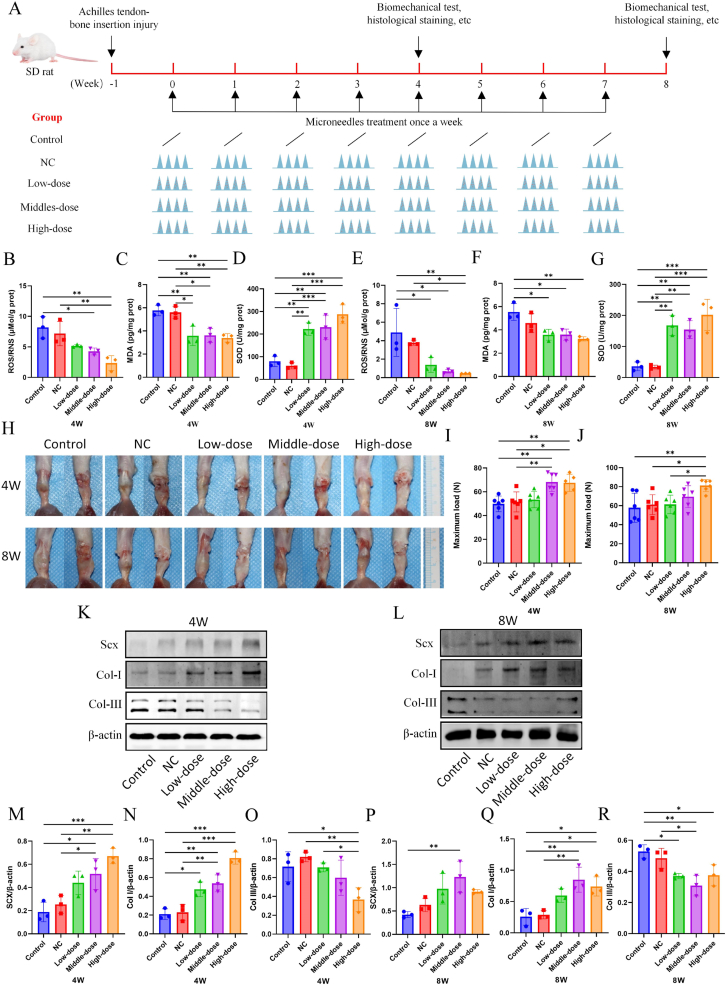
**DMN/PLGA@Dia alleviated oxidative stress during tendon-bone insertion healing and increased the strength of tendon-bone insertion healing.** (A) Timeline of experiments for rat model. (B–D) The relative level of ROS/RNS, MDA and SOD in tendon-bone insertion tissues at 4 W (n = 3). (E–G) The relative level of ROS/RNS, MDA and SOD in tendon-bone insertion tissues at 8 W (n = 3). (H) Typical images of the repaired Achilles tendon-bone tissues in different groups at 4 and 8 W. (I) Maximum load of Achilles tendon-bone tissues in different groups at 4 W (n = 6). (J) Maximum load of Achilles tendon-bone tissues in different groups at 8 W (n = 6). (K) Expression of SCX, Col I and Col III of Achilles tendon-bone tissues in different groups at 4 W. (L) Expression of Col I, Col III and SCX of Achilles tendon-bone tissues in different groups at 8 W. (M − O) Representative western blot assay of SCX, Col I and Col III of Achilles tendon-bone tissues in different groups at 4 W (n = 3). (P–R) Western blot assay of SCX, Col I and Col III of Achilles tendon-bone tissues in different groups at 8 W (n = 3). ∗P < 0.05; ∗∗P < 0.01; ∗∗∗P < 0.001.

To ascertain the efficacy of DMN/PLGA@Dia in enhancing regenerative tendon-bone repair in vivo, leveraging earlier studies, we developed a rat model with Achilles tendon-bone injuries [43]. The role of DMN/PLGA@Dia in combating oxidative stress is first investigated. Fig. 5A shows the timeline of experiments for rat model. At four-week and eight-week, the DMN/PLGA@Dia groups exhibit a sustained reduction in ROS/RNS levels compared to both the Control and NC groups, with the reduction being most pronounced in the High-dose group (P < 0.01) (Fig. 5B and E). MDA, a maker of lipid peroxidation damage marker in mitochondria, also improves significantly in DMN/PLGA@Dia groups (Fig. 5C and F). Antioxidant enzymes serve as the primary safeguard against ROS, shielding the mitochondrial contents and reinforcing the stability of mitochondrial membranes [44]. SOD serves as the primary safeguard against oxidative stress. Our findings revealed a notable resurgence in SOD levels within the DMN/PLGA@Dia treatment groups (Fig. 5D and G). Fig. 5H shows the Achilles tendon-bone in five groups at weeks 4 and 8. From the overall picture, it can be observed that the Achilles tendon-bone tissues in DMN/PLGA@Dia treated groups are significantly milky white with less adhesion, which is similar to normal Achilles tendon-bone tissues. The Achilles tendon-bone tissues in the Control group and NC group exhibits transparency and severe adhesion. For biomechanical tests, all tissues are ruptured at the injury site. At weeks 4, the Middle-dose DMN/PLGA@Dia group and the High-dose DMN/PLGA@Dia group has significantly higher maximum loads than the Control group and NC group (Fig. 5I). By the eighth week, the High-dose DMN/PLGA@Dia group alone exhibits a notably greater maximum load when compared to both the Control and NC groups (Fig. 5J). This may be because the adhesion in the Control group and the NC group is heavier, and these scar tissues can also improve the maximum load to some extent.

To measure the effect of DMN/PLGA@Dia on the Achilles tendon-bone tissues, the protein levels of the transcription factor SCX, Col I and Col III are tested by WB at weeks 4 (Figs. 5K) and 8 (Fig. 5L). SCX is expressed throughout the development of tendons and plays a crucial role in guiding tendon wound healing [45]. The restoration of the tendon-bone interface's microstructure plays a crucial role in the healing process of this junction [[46], [47], [48]]. At the interface where tendon meets bone, Col I and Col III fulfill distinct yet mutually supportive functions, contributing significantly to the restoration of the tendon-bone connection, particularly during the critical phase of integration following injury or surgical intervention. Col I serves as the principal architectural collagen within tendons and skeletal structures, endowing them with tensile fortitude and resilience against elongation. Its significance intensifies during the latter phase of the healing process, where it facilitates the development of a robust, fibrous boundary capable of withstanding mechanical pressures. As the tissue matures, the concentration of Col I increases, aligning in the direction of stress to enhance the mechanical robustness of the repaired area. Col III is more abundant in the early stage of tendon-bone healing. It plays a crucial role in forming a more adaptable temporary matrix, capable of swiftly filling the affected area and offering support for the initial cellular invasion. This kind of collagen serves as a framework for cells and various extracellular matrix elements, fostering cellular movement and the preliminary structuring of the tissue [49]. As healing progresses, Col III is gradually replaced by Col I as the tissue becomes more organized and stronger [50]. This shift is pivotal to the renovation and maturation of the healing area. The two forms of collagen play a vital role in the creation of the tendon-bone junction, yet the equilibrium between them is of utmost importance. During the initial phase of healing, elevated concentrations of Col III are instrumental in fostering cellularity and vascularity, thereby facilitating the preliminary tissue restoration and diminishing scar tissue development. Col I plays a crucial role in affording the necessary durability and strength for sustained stability over the long term. Any imbalance between Col I and III has the potential to compromise the healing process, resulting in tissues that are either fragile or excessively fibrotic, and which consequently may not bear mechanical stresses with the required resilience [28,51]. In our results, at week 4 and week 8, the expression of SCX and Col I is significantly boosted in DMN/PLGA@Dia treated groups compared with the Control and NC groups (Fig. 5M, N, 5P and 5Q). At week 4 and week 8, the expression of Col III is significantly decreased in the DMN/PLGA@Dia treated groups compared with the Control and NC groups (Fig. 5O and R).

**Fig. 6:**
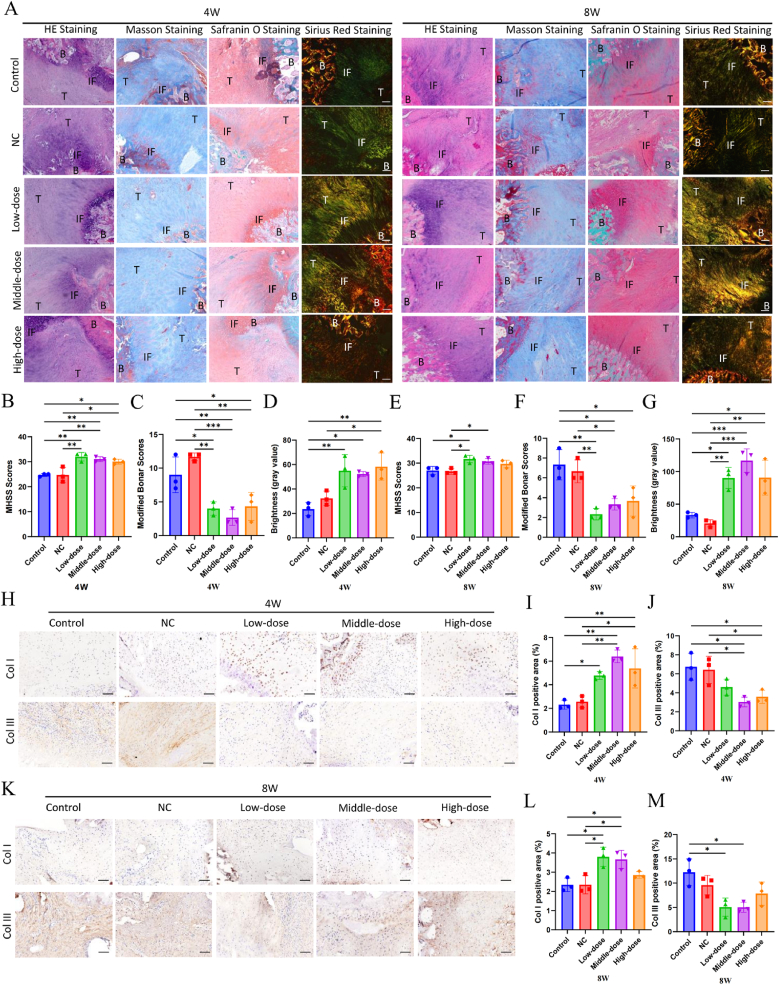
**DMN/PLGA@Dia promoted the healing of tendon-bone insertion tissues.** (A) Typical H&E staining, Masson's staining, Safranin O staining and Sirius red staining images of Achilles tendon-bone point tissues in different groups at 4 weeks and 8 weeks (Sirius red staining, coarse orange or bright red fibre was Col I, while fine green fibre was Col III or fibrochondrocyte). Scale bars = 100 μm. (B) The TMSS for the histological evaluation of Achilles tendon-bone point tissues in different groups at 4 weeks (n = 3). (C) The modified Bonar scores for the histological evaluation of Achilles tendon-bone point tissues in different groups at 4 weeks (n = 3). (D) Semi-quantitative analysis of the Col I content in Achilles tendon-bone point tissues was based on brightness at 4 weeks (n = 3). (E) The TMSS for the histological evaluation of Achilles tendon-bone point tissues in different groups at 8W (n = 3). (F) The modified bonar scores for the histologic evaluation of Achilles tendon-bone point tissues in different groups at 8 weeks (n = 3). (G) Semi-quantitative analysis of the Col I content in Achilles tendon-bone point tissues was based on brightness at 8W (n = 3). (H) Typical immunohistochemical staining images of Col I and Col III in Achilles tendon-bone point tissues at 4 weeks. Scale bars = 100 μm (I) Semi-quantitative analysis of the Col I content in Achilles tendon-bone point tissues at 4 weeks (n = 3). Scale bars = 100 μm (J) Semi-quantitative analysis of the Col III content in Achilles tendon-bone insertion tissues at 4 weeks (n = 3). (K) Typical immunohistochemical staining images of Col I and Col III in Achilles tendon-bone point tissues at 8W. Scale bar = 100 μm (I) Semi-quantitative analysis of the Col I content in Achilles tendon-bone point tissues at 8W (n = 3). (J) The semiquantitative analysis of the Col III content in Achilles tendon-bone point tissues at 8W (n = 3). ∗P < 0.05; ∗∗P < 0.01; ∗∗∗P < 0.001. (For interpretation of the references to colour in this figure legend, the reader is referred to the Web version of this article.)

HE and Masson staining results show that the junction tissue in DMN/PLGA@Dia at weeks 4, each group is organized and crossed over. In contrast, the collagen tissue distribution in the Control group and NC group is sparse and irregularly adhered to the insert. At 8 weeks, the orientation of collagen fibers at the implantation sites in each group are more regular than in the early stages (Fig. 6A). Particularly, the insertion sites in the Low-dose and Medium-dose DMN/PLGA@Dia Groups exhibit a higher degree of organization compared to the other groups. In the semi-quantitative analysis, the MHSS scores for both the Control group and the NC group were substantially lower than those recorded for the DMN/PLGA@Dia treated groups at both weeks 4 and weeks 8 (Fig. 6B and D). At 4 weeks, Safranin O staining yields evidence that the chondrocytes within the DMN/PLGA@Dia scaffold in each group exhibit a more advanced state of maturity, with their differentiation from fibroblasts proceeding in a more organized manner. This trend intensifies and becomes even more evident by the eighth week (Fig. 6A). Four weeks post-surgery, the Sirius red staining of tissue sections reveals that the collagen within DMN/PLGA@Dia exhibits a more uniform and organized structure, in contrast to the Control and NC groups (Fig. 6A). A preliminary quantitative assessment, utilizing the intensity of Sirius Red staining at 4-week intervals, demonstrates that the Col-I content in the control group was notably inferior compared to that observed in the DMN/PLGA@Dia treated groups (Fig. 6D). At 8 weeks, the content of Col-I in DMN/PLGA@Dia treated groups are significantly greater than the Control group and NC group (Fig. 6G). At 4 weeks, immunohistochemical results displays that the expression of Col-I in the Control group and NC group is significantly lower than that in the DMN/PLGA@Dia group (Fig. 6H and I). The Col III expression in both the Control and NC groups is markedly elevated compared to the Medium-dose DMN/PLGA@Dia and High-dose DMN/PLGA@Dia groups (Fig. 6H and J). At 8 weeks, immunohistochemical analysis reveals that the Col I expression in both the Control and NC groups is markedly diminished compared to the Low-dose DMN/PLGA@Dia and Middle-dose DMN/PLGA@Dia groups (Fig. 6K and L). The expression of Col III in the Control group is considerably higher than that in the Low-dose DMN/PLGA@Dia group and Middle-dose DMN/PLGA@Dia groups (Fig. 6K and M). The results obtained from immunohistochemistry are in line with those from Western blot analysis. Given the significance of Col I and Col III in tendon-bone healing, it is speculated that Dia may promote healing through two mechanisms [52]. Firstly, Dia has the potential to enhance the early neovascularization of Col III, and secondly, it may expedite the transformation of Col III into Col I. These processes collectively contribute to the facilitation of tendon-bone union. It is thought that the second possibility is more likely due to the decrease in Col III content at 4 weeks. Conversely, a period of four weeks is generally not regarded as an initial phase in the healing process of tendons and bones. Hence, additional studies are essential to substantiate this claim.

### 3.6 *In vivo* DMN/PLGA@Dia improved functional recovery after achilles tendon insertion injury

**Fig. 7:**
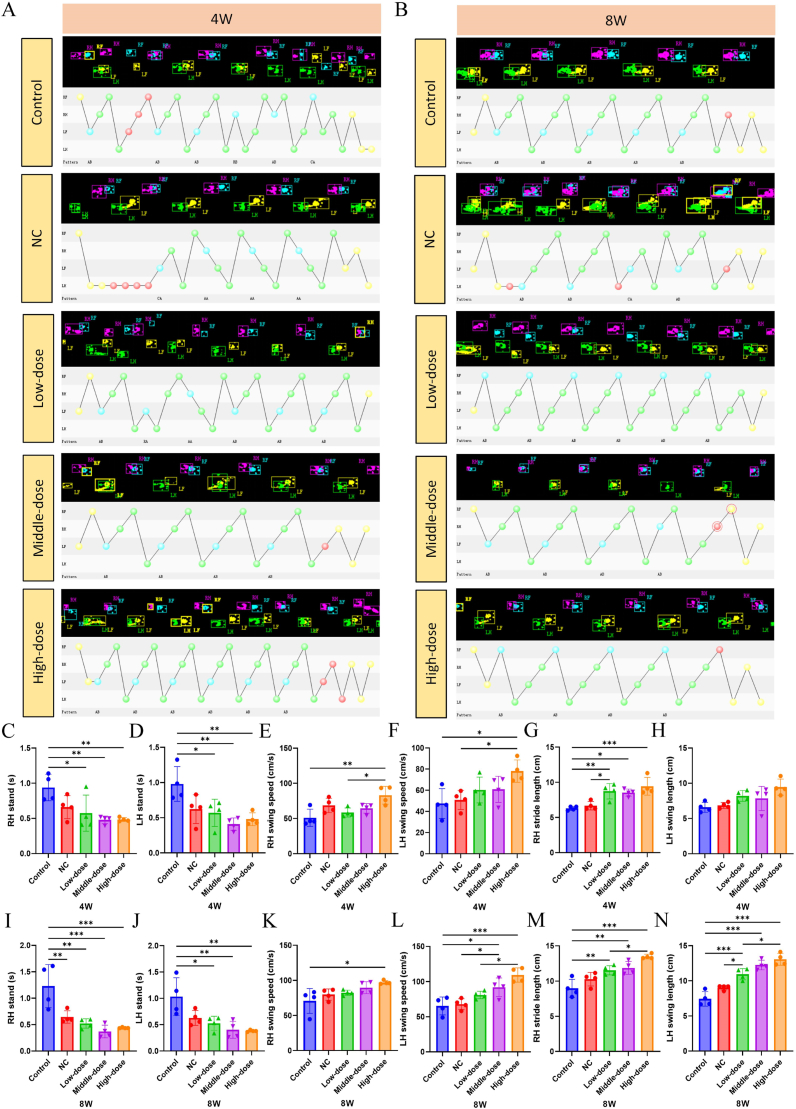
**DMN/PLGA@Dia improved functional recovery after Achilles tendon insertion injury.** (A) Representative CatWalk footprint images and analysis of different groups at 4 weeks. (B) Representative CatWalk footprint images and analysis of different groups at 8 weeks. (C–H) Three parameters of the footprint analysis were used to quantify the hind limb function. (C–D) Stance. (E–F) Swing speed. (G–H) Swing length of different groups at 4 weeks (n = 4). (I–N) Three parameters of footprint analysis were used to quantify the hind limb function. (I–J) Stance. (K–L) Swing speed. (M − N) Swing length of different groups at 8 weeks (n = 4). ∗P < 0.05; ∗∗P < 0.01; ∗∗∗P < 0.001.

To achieve a precise and unbiased assessment of the additional DMN/PLGA@Dia characteristics, such as gait stability, the CatWalk analysis is conducted on the rodents across various experimental groups (Fig. 7 and Fig. S8). The outcomes suggest that by 4 and 8 weeks, the groups treated with DMN/PLGA@Dia displayed a marked inclination toward exhibiting normal hind limb footprints in comparison to the Control group (Fig. 7A and B). Swing speed, stride length and length are typically used indicators to assess gait [53]. At 4 weeks, most of the DMN/PLGA@Dia treated groups significantly improve the swing speed, and stride length and decreases the stance time during walking of the model rats compared to those of the Control group (Fig. 7C–H). At 8W, most of the different DMN/PLGA@Dia groups significantly boost the swing speed (RH and LH) and stride length (RH and LH), as well as decrease the stance time during walking (RH and LH) of the model rats compared with those of the Control group (Fig. 7I–N). Furthermore, prior research has indicated that robust rats tend to avoid placing their heels flat on the ground throughout the walking cycle, a behavior not exhibited by those rats suffering from Achilles tendon damage [54,55]. In our investigation, representative footprint images demonstrate that at both 4-week and 8-week intervals, the rats in the control group maintained full heel contact with the ground. Conversely, the rats treated with DMN/PLGA@Dia exhibit the capability to execute heel lift actions. The findings are consistent with prior studies. In addition, the paw strain graphs at both 4-week and 8-week intervals display that the rats in the treatment group exhibited enhanced tolerance to strain (Fig. S8). All these results suggest that DMN/PLGA@Dia promotes the functional recovery after Achilles tendon-bone insertion injury.

### 3.7 Dia adjusted macrophage M1/M2 polarization in vivo

**Fig. 8:**
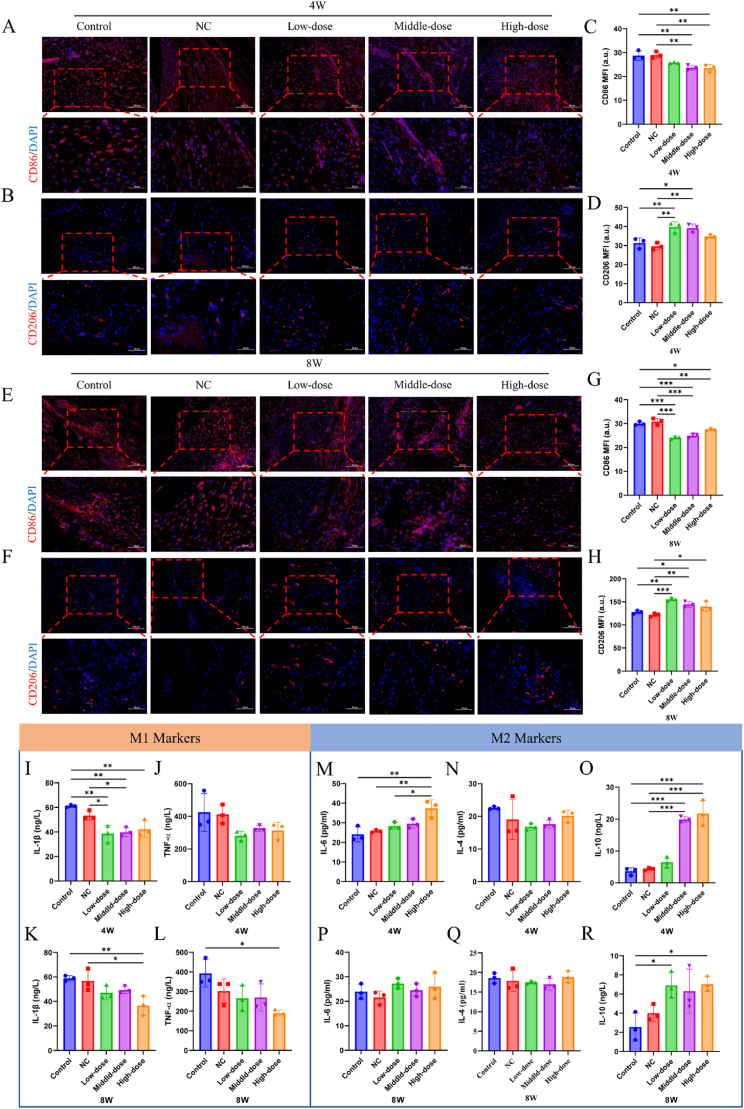
**DMN/PLGA@Dia adjusted macrophage M1/M2 polarization in vitro.** (A) Typical fluorescence images of cell nucleus (DAPI, blue) with CD86 (red) in Achilles tendon-bone point tissues at 4 weeks. Scale bars = 100 μm (B) Typical fluorescence images of cell nucleus (DAPI, blue) with CD206 (red) in Achilles tendon-bone point tissues at 4 weeks. Scale bars = 100 μm (C) The semiquantitative analysis of the CD86 content in Achilles tendon-bone insertion tissues at 4 weeks (n = 3). (D) Semi-quantitative analysis of the CD206 content in Achilles tendon-bone insertion tissues at 4 weeks (n = 3). (E) Typical fluorescence images of cell nucleus (DAPI, blue) with CD86 (red) in Achilles tendon-bone insertion tissues at 8W. Scale bars = 100 μm (F) Typical fluorescence images of cell nucleus (DAPI, blue) with CD206 (red) in Achilles tendon-bone point tissues at 8W. Scale bars = 100 μm (G) The semiquantitative analysis of the CD86 content in Achilles tendon-bone insertion tissues at 8W (n = 3). (H) The semiquantitative analysis of the CD206 content in Achilles tendon-bone insertion tissues at 8W (n = 3). (I–L) The expression of cytokines related to M1 macrophage phenotype was detected by ELISA, including IL-1β (I, K) and TNF-α (J, L) at 4 weeks and 8W (n = 3). (M–R) Expression of cytokines related to M2 macrophage phenotype was detected by ELISA, including IL-6 (M, P), IL-4 (N, Q) and IL-10 (O, R) at 4 weeks and 8W (n = 3). ∗P < 0.05; ∗∗P < 0.01; ∗∗∗P < 0.001. (For interpretation of the references to colour in this figure legend, the reader is referred to the Web version of this article.)

The healing of tendon-to-bone interfaces is a complex biological process involving inflammation, extracellular matrix remodeling, and tissue regeneration. Macrophages, as key orchestrators of the immune response, play a crucial role in regulating these phases through their polarization into pro-inflammatory (M1) and anti-inflammatory/pro-regenerative (M2) phenotypes. Several studies have explored this relationship between the polarization of macrophages and tendon-bone healing. For instance, the study on MFG-E8 highlights its role in promoting tendon-bone healing by regulating macrophage efferocytosis and M2 polarization after anterior cruciate ligament reconstruction (ACLR). The research demonstrated that MFG-E8 facilitates the clearance of apoptotic cells and increases the number of M2 macrophages at the tendon-bone interface, which is crucial for effective healing [56]. Similarly, mechanical stimulation has been shown to improve tendon-bone healing by activating the IL-4/JAK/STAT signaling pathway, which mediates macrophage M2 polarization. This pathway's activation enhances the quality of tendon-bone healing, as observed in a rotator cuff repair model, indicating the potential of mechanical cues in modulating macrophage behavior to favor healing [57]. Moreover, bone marrow stromal cell-derived exosomes (BMSC-Exos) have been identified as a promising therapeutic strategy for tendon-bone healing. These exosomes promote the polarization of macrophages from the M1 to the M2 phenotype, thereby reducing the early inflammatory reaction at the tendon-bone interface and promoting healing after ACLR. The presence of specific miRNAs, such as miR-23a-3p, in BMSC-Exos plays a crucial role in this polarization process, highlighting the potential of exosome-based therapies in enhancing tendon-bone healing [58]. These studies collectively underscore the importance of macrophage polarization in tendon-bone healing and suggest that therapeutic strategies aimed at promoting M2 polarization could significantly enhance healing outcomes. By understanding and manipulating the macrophage polarization process, it may be possible to develop more effective treatments for tendon-bone injuries. In this research, the regulatory role of DMN/PLGA@Dia on the immune microenvironment during tendon-bone insertion healing is explored. Pathologically, oxidative stress mediates inflammatory activation, including the activation of M1 macrophages and unrestricted secretion of pro-inflammatory factors [59,60]. Therefore, during the intervention of DMN/PLGA@Dia in the tendon-bone healing process, the direction of macrophage polarization and the secretion of inflammatory factors are investigated. For the purpose of macrophage polarization analysis, M1 macrophages are labeled with CD86 and M2 macrophages are labeled with CD206 for tissue immunofluorescence staining. The quantitative comparison between 4 and 8 weeks indicates that the intervention of DMN/PLGA@Dia intervention resulted in a significantly lower proportion of CD86 positive cells compared to the Control group and NC group, and a significantly higher proportion of CD206 positive cells compared to the Control group and NC group (Fig. 8A–H). This indicated that the development of macrophage polarization towards M2. Subsequently, the secretion of inflammatory and anti-inflammatory factors in the tendon-bone tissue is detected at 4 and 8 weeks. The ELISA results display a significant decrease in the secretion of the inflammatory factor IL-1β in the DMN/PLGA@Dia treated groups at 4 and 8 weeks. TNF-α shows a decreasing trend. IL-6 and IL-10 secretion increased significantly (Fig. 8I–R). Not like IL-1β, TNF-α and IL-10, IL-6 is a pleiotropic cytokine [61]. In the nascent phase of the IL-6 commonly acts as a pro-inflammatory agent, intensifying the inflammatory reaction and contributing to tissue injury. However, as the condition reaches a comparative plateau or enters a state of remission subsequent to efficacious therapy, IL-6 potentially teams up with IL-10 to facilitate the transition of macrophages from the pro-inflammatory M1 type to the anti-inflammatory M2 type [62,63]. In our study, the two time points of 4W and 8W are not considered early stages of the disease for tendon-bone healing. Therefore, IL-6 will be incorporated as one of the markers for M2 polarization. These findings collectively suggest that the shift in the tendon-bone insertion's healing immune microenvironment from a pro-inflammatory to an anti-inflammatory state is activated by the immunomodulatory influence of DMN/PLGA@Dia.

Finally, the biocompatibility of DMN/PLGA@Dia is verified by HE staining of organs, blood biochemical tests and haemolysis tests. The results indicate no significant pathological changes of major organs (Fig. S9A–B). Furthermore, the blood biochemical indices exhibited no discernible variation between the DMN/PLGA@Dia groups and the control group (Fig. S9C–T). The haemolysis rate of PLGA@Dia is less than 5% (Fig. S10).

## 4 Conclusion

Our research indicates that DMN/PLGA@Dia can be employed as an effective and universal treatment method to foster tendon bone insertion repair and significantly improve the immune microenvironment. The therapeutic benefits and efficacy of DMN/PLGA@Dia are systematically studied. By shielding tendon-bone cells from ROS-induced damage, the regeneration of tendon-bone junctions can be notably expedited. Additionally, the findings of the study affirm that DMN/PLGA@Dia demonstrates the capability to proficiently counteract detrimental immune responses by facilitating the transition of macrophages from a pro-inflammatory M1 phenotype to an anti-inflammatory M2 phenotype. In addition, using a rat Achilles tendon bone injury model, it is validated that a simple DMN/PLGA@Dia can enhance functional recovery. This report describes an effective nanomedicine delivery system for tendon bone insertion repair. Therefore, it is believed that our research will provide an effective strategy for tendon-bone insertion repair.

## CRediT authorship contribution statement

**Jie Sun:** Writing – original draft, Methodology, Formal analysis, Data curation, Conceptualization. **Qing Zhong Chen:** Software, Methodology, Formal analysis, Data curation. **Ai Zi Hong:** Software, Methodology, Investigation, Formal analysis, Data curation. **Fei Ju:** Methodology, Formal analysis, Data curation. **Hao Liang Wang:** Software, Methodology. **Bo Zhang:** Software, Methodology. **Wang Liu:** Methodology. **Yu Cheng Sun:** Software, Methodology. **Jun Tan:** Visualization, Supervision, Project administration, Investigation, Funding acquisition, Formal analysis. **Qian Qian Yang:** Writing – review & editing, Software, Resources, Project administration, Methodology, Investigation. **You Lang Zhou:** Writing – review & editing, Validation, Project administration, Funding acquisition, Data curation, Conceptualization.

## Ethics approval

The experiment protocol was approved by the Experimental Animal Care and Use Committee of Nantong University (code S20240420-191).

## Declaration of competing interest

The authors declare that they have no known competing financial interests or personal relationships that could have appeared to influence the work reported in this paper.

## Data Availability

Data will be made available on request.

## Abbreviations

Dia: diacerein
DMN: dissolvable microneedle
PDMS: Polydimethylsiloxane
ROS: Reactive oxygen species
PLGA: poly lactic-co-glycolic acid
LPS: lipopolysaccharide
H&E: Hematoxylin and Eosin
Dir: 1,1-dioctadecyl-3,3,3,3-tetramethylindotricarbocyaine iodide
FBS: fetal bovine serum
GEO: Gene Expression Omnibus
NCBI: National Center for Biotechnology Information
DEGs: differentially expressed genes
TPM: Transcripts Per Million
logFC: log2 fold change
SEM: scanning electron microscopy
TEM: transmission electron microscopy
DLS: dynamic light scattering
PCA: Principal component analysis
CCK-8: Cell Counting Kit-8
SDS: sodium dodecyl sulfate
PVDF: polyvinylidene fluoride
PDMS: Polydimethylsiloxane
IACUC: Institutional Animal Care and Use Committee
*β*-gal: beta-galactosidase
SCX: sclerosis
