# Supplementary material for: Dissolvable microneedle-assisted transdermal administration of diacerein nanoparticles achieved satisfactory therapeutic effects in tendon-bone insertion repair by reducing oxidative stress and inflammation
Source: Mater Today Bio. 2025 Jun 18;33:101999. doi: 10.1016/j.mtbio.2025.101999 (PMC12240096; doi:10.1016/j.mtbio.2025.101999)
Supplement: Multimedia component 1 [file mmc1.docx]

**Supplementary file**

**Dissolvable microneedle-assisted transdermal administration of diacerein nanoparticles achieved satisfactory therapeutic effects in tendon-bone insertion repair by reducing oxidative stress and inflammation**

Jie Sun^1^, Qing Zhong Chen^1^, Ai Zi Hong^1^, Fei Ju, Hao Liang Wang, Bo Zhang, Wang Liu, Yu Cheng Sun, Jun Tan^#^, Qian Qian Yang^#^, You Lang Zhou^#^

Hand Surgery Research Center, Research Center of Clinical Medicine, Affiliated Hospital of Nantong University, Medical School of Nantong University, Nantong 226001, China.


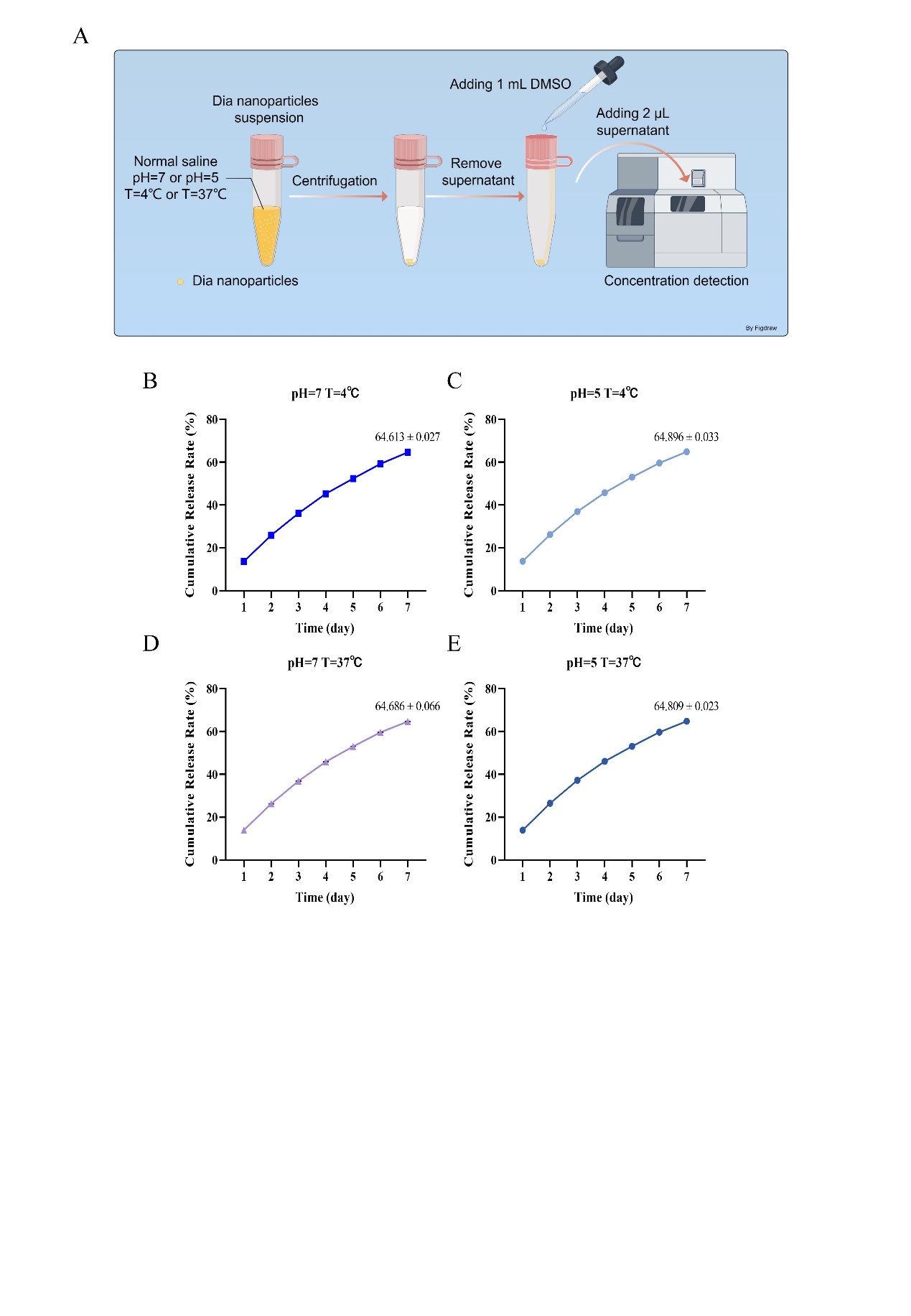


**Figure S1. In vitro release rate of Dia form Dia nanoparticles in in two different PH and two different temperatures.** (A) Schematic diagram of the process for detecting the daily release of Dia form Dia nanoparticles. (B) The release rate of Dia form Dia nanoparticles at a pH of 7 and a temperature of 4 degrees within 7 days. (C) The release rate of Dia form Dia nanoparticles at a pH of 5 and a temperature of 4 degrees within 7 days. (D) The release rate of Dia form Dia nanoparticles at a pH of 7 and a temperature of 37 degrees within 7 days. (E) The release rate of Dia form Dia nanoparticles at a pH of 5 and a temperature of 37 degrees within 7 days.


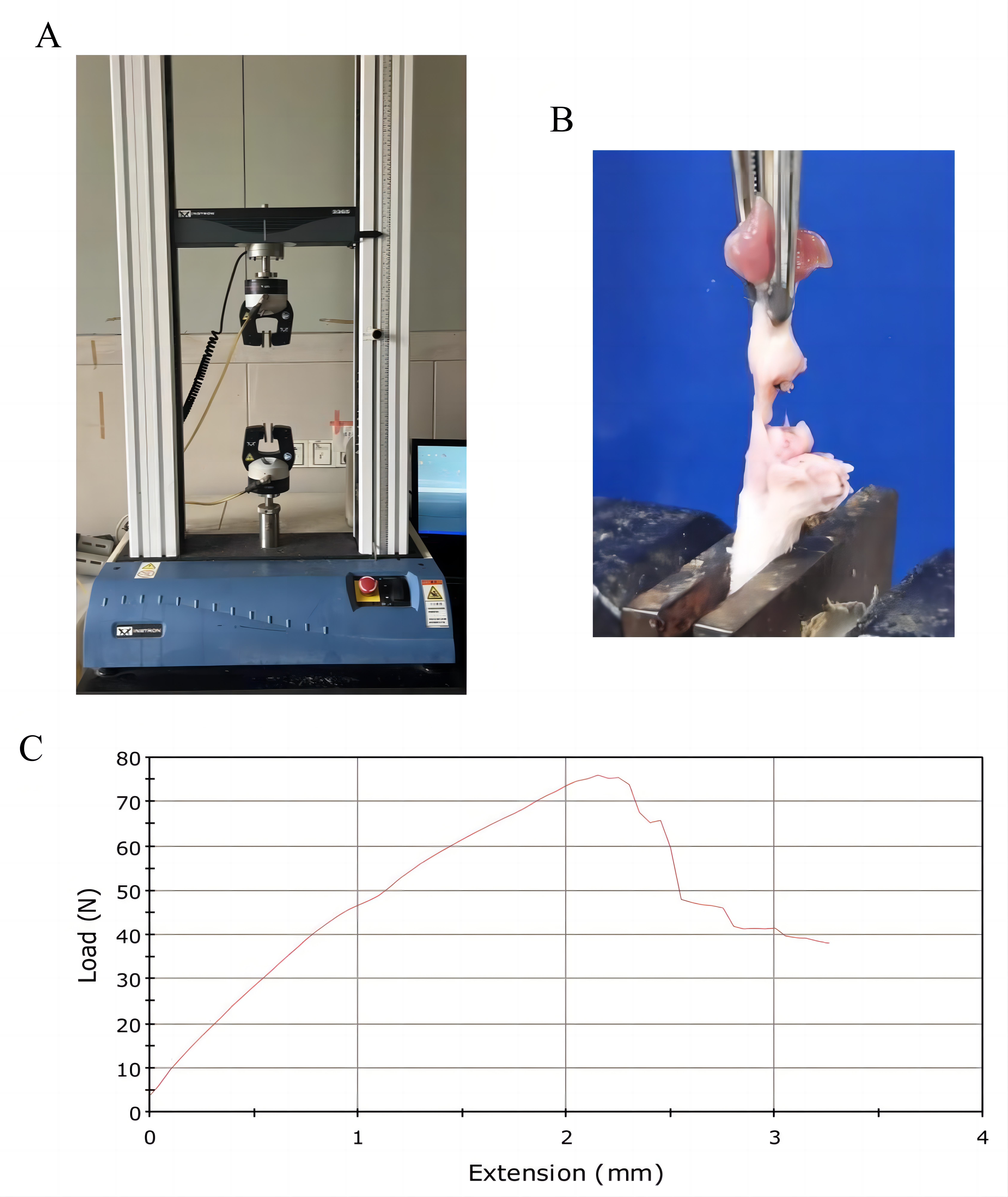


**Figure S2. Biomechanical testing of the repaired Achilles tendon-bone insertion.** (A) Instron testing machine (model 3365; Instron Corp.). (B) Both ends of the Achilles tendon were clamped in the lower and upper clamps, with the repair site held in the middle of the two clamps. The upper clamp holding one end of the tendon was pulled at a constant speed of 25 mm/min and stopped when the Achilles tendon-bone tip ruptured. (C) Typical maximum tension diagram measured by testing software program (Series IX; Instron).


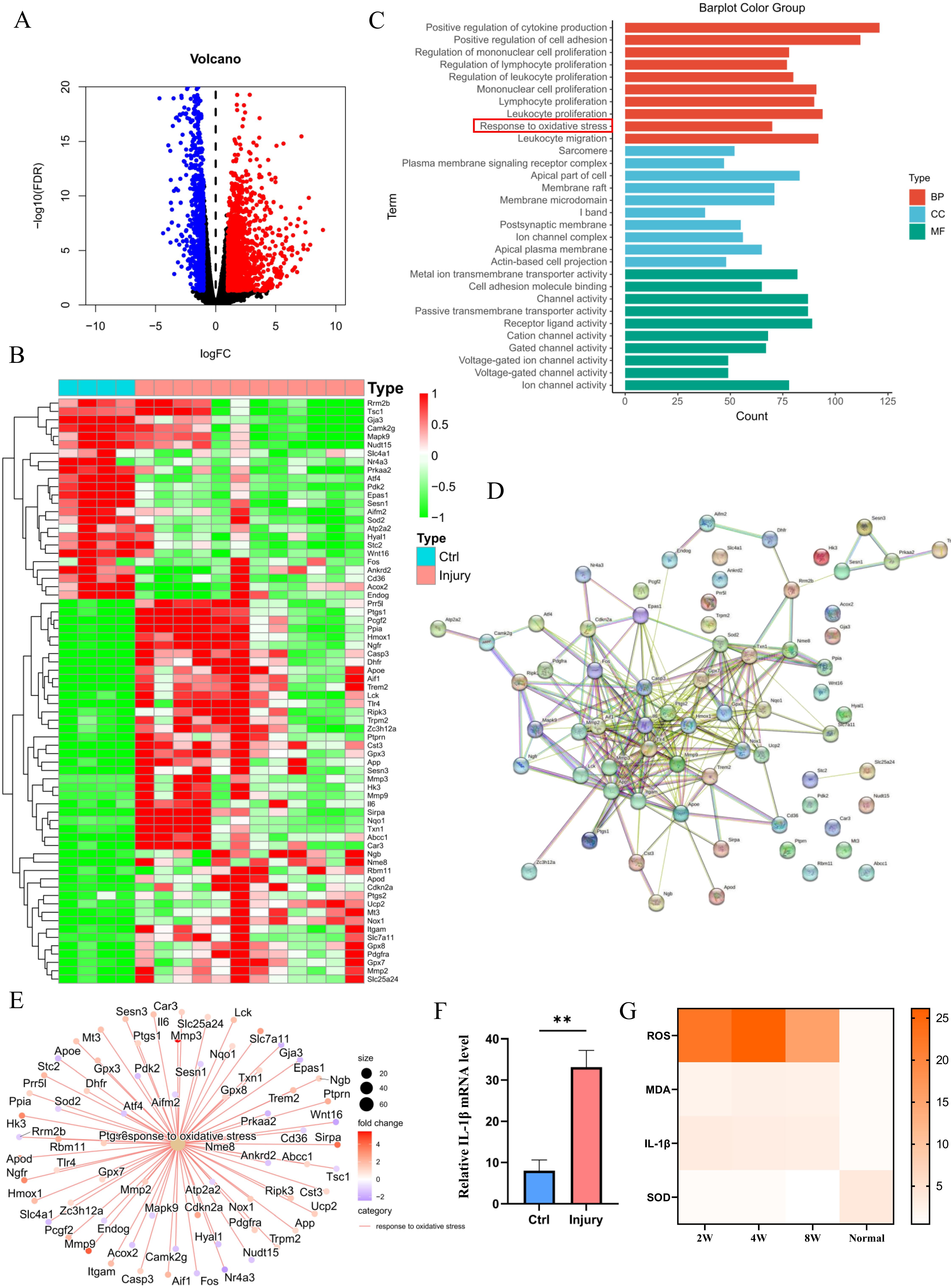


**Figure S3.** **Persistent oxidative stress in tendon-bone tissue at different time points after injury.** (A) Volcano diagram of DEGs. (B) Heat map of DEGs. (C) GO functional enrichment analysis of DEGs. (D) PPI protein network analysis of DEGs. (E) KEGG analysis of DEGs. (F) The mRNA expression level of IL-1β in the expression matrix. (G) ELISA results of ROS/RNS, MDA, IL-1β, and SOD levels of normal rats and different time points after tendon-bone insertion injury (for ROS/RNS, MDA and IL-1β, normal as a reference; for SOD, 2W group as a reference). **P<0.01.


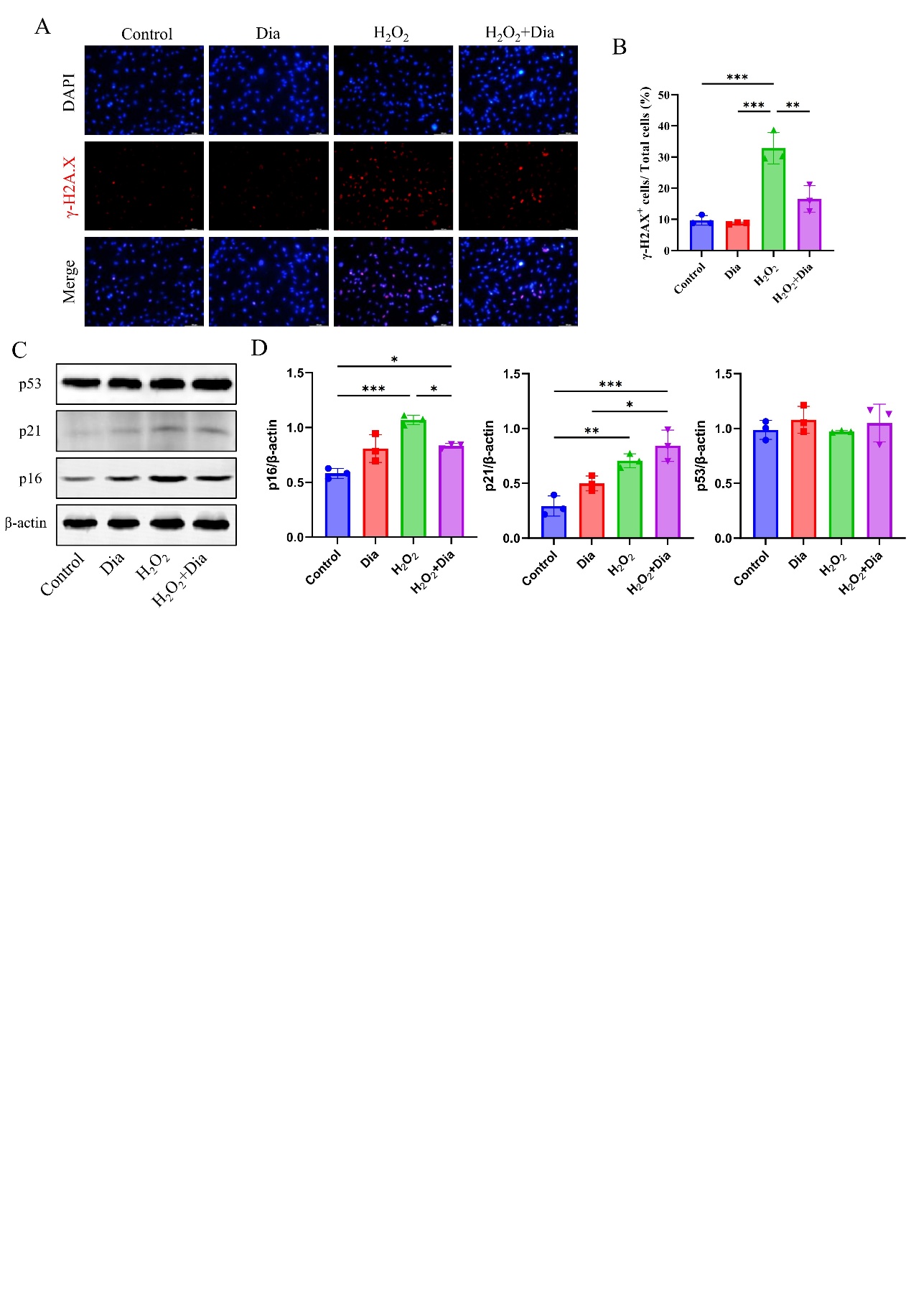


**Figure S4.** **Dia protects against oxidative stress-induced DNA damage and cellular senescence.** (A) Representative immunofluorescence images of γ-H2A.X staining. (B) Quantification of γ-H2A.X staining (n=3). (C) Western blot analysis of senescence markers: p16, p21 and p53. (D) Western blot assay of p16, p21 and p53 (n=3). *P<0.05; **P<0.01; *** P<0.001.


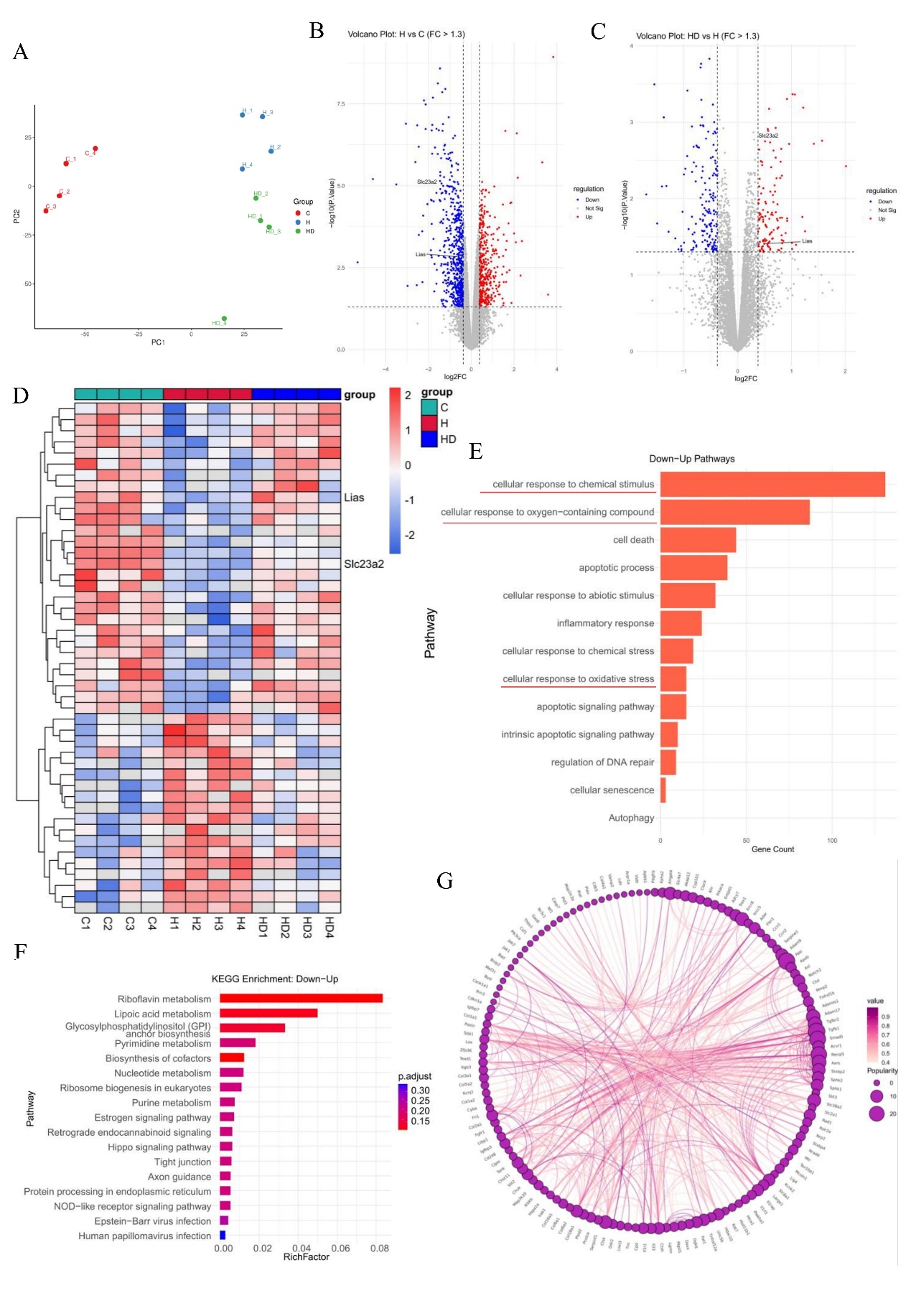


**Figure S5. Proteomic analysis of different tendon cells groups.** (A) Principal component analysis of three groups. (B) Differential protein volcano plot of H_2_O_2_ group versus Control group. (C) Differential protein volcano plot of H_2_O_2_ + Dia group versus H_2_O_2_ group. (D) Differential expression heatmap of overlapping proteins (intersection of down regulated proteins in figure B and up regulated proteins in figure C, intersection of up regulated proteins in figure B and down regulated proteins in figure C). (E) GO functional enrichment analysis of intersecting proteins, based on the intersection of down regulated proteins in figure B and up regulated proteins in figure C. (F) KEGG functional enrichment analysis of intersecting proteins, based on the intersection of down regulated proteins in figure B and up-regulated proteins in figure C. (G) PPI protein network interaction analysis of intersecting proteins.


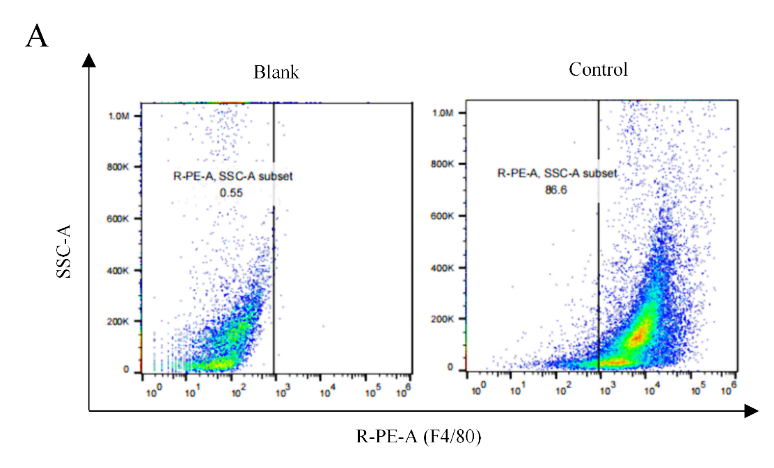


**Figure S6.** **Flow cytometry identification of primary peritoneal macrophages.** (A) Flow cytometry staining results of rat macrophage markers F4/80.


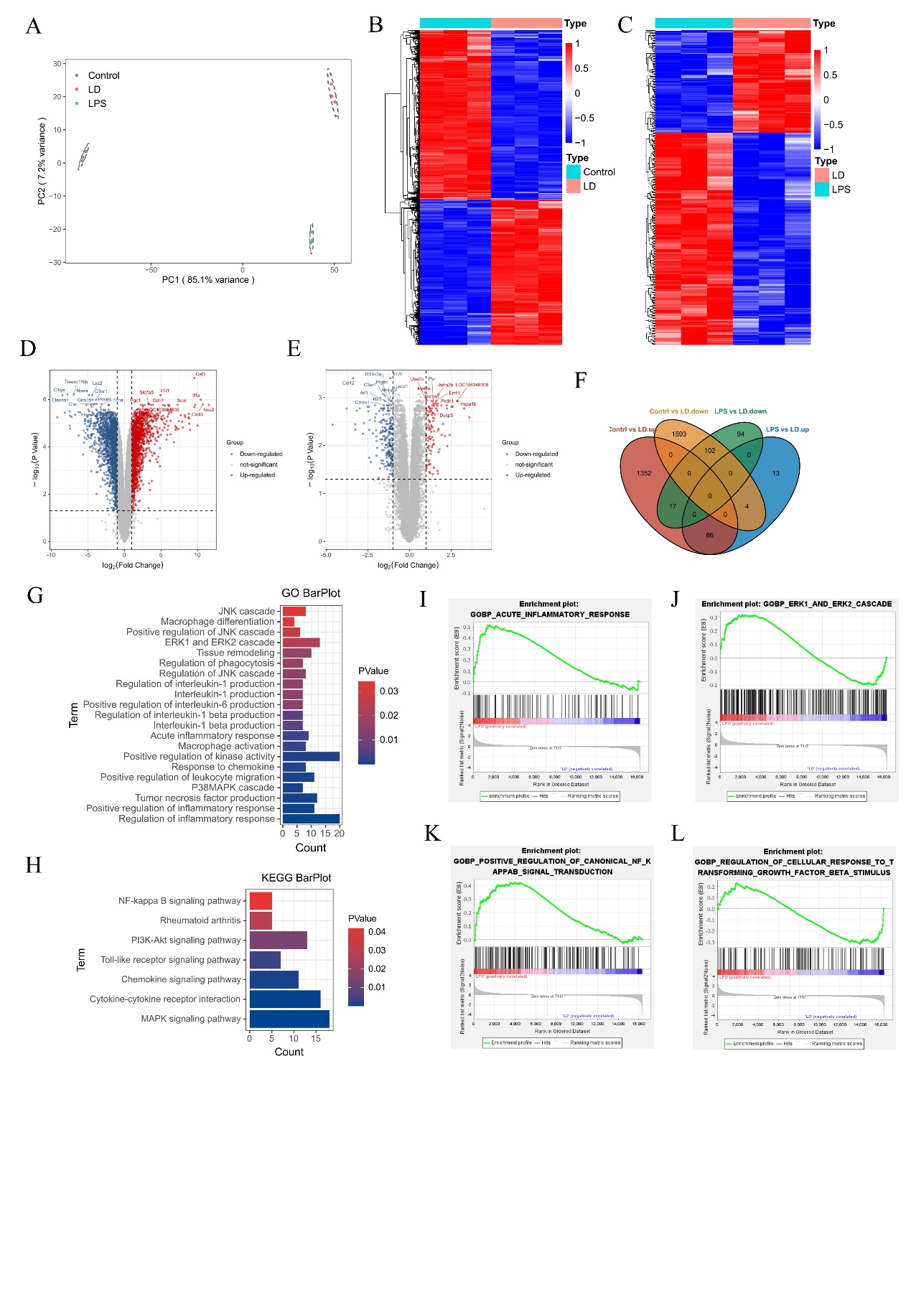


**Figure S7. RNA-Sequencing result of different macrophage groups.** (A) Principal component analysis of three groups. (B-E) Differentially gene expression heatmaps and volcano plots of different groups. (F) Venn analysis of common differentially expressed genes in the LPS+Dia group. (G) Biological process of GO enrichment analysis. (H) KEGG enrichment analysis. (I-L) GSEA analysis of the activation or inhibition for the acute inflammation pathway, NF-κB signaling cascade, ERK signaling and TGF-β signaling in LPS+Dia group.


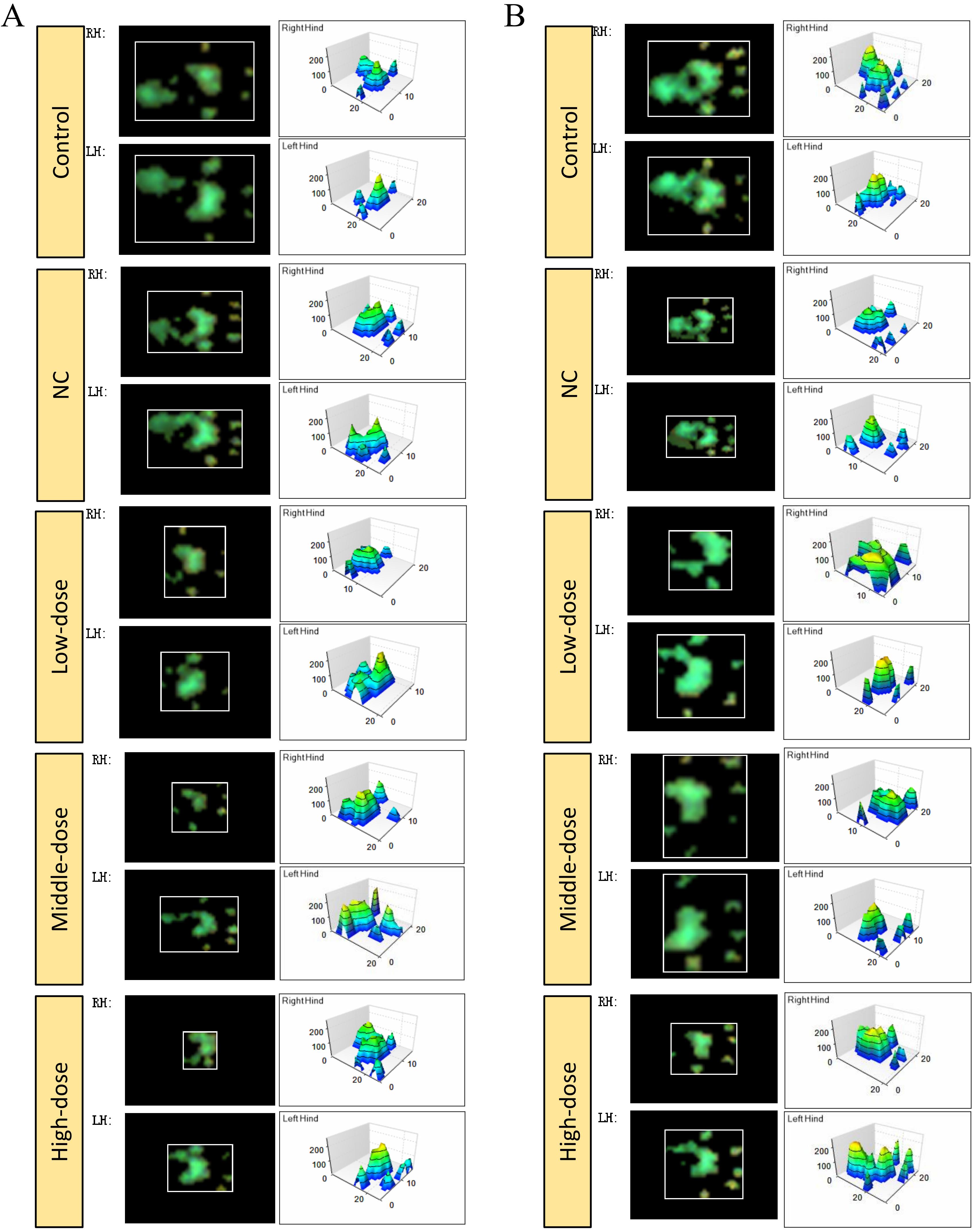


**Figure S8. DMN/PLGA@Dia promoted the recovery of heel lifting movements and increased the maximum load borne.** (A) Representative CatWalk hind paw footprint images of different groups and stress diagram at 4 weeks. (B) Representative CatWalk hind paw footprint images of different groups and stress diagram at 8 weeks.


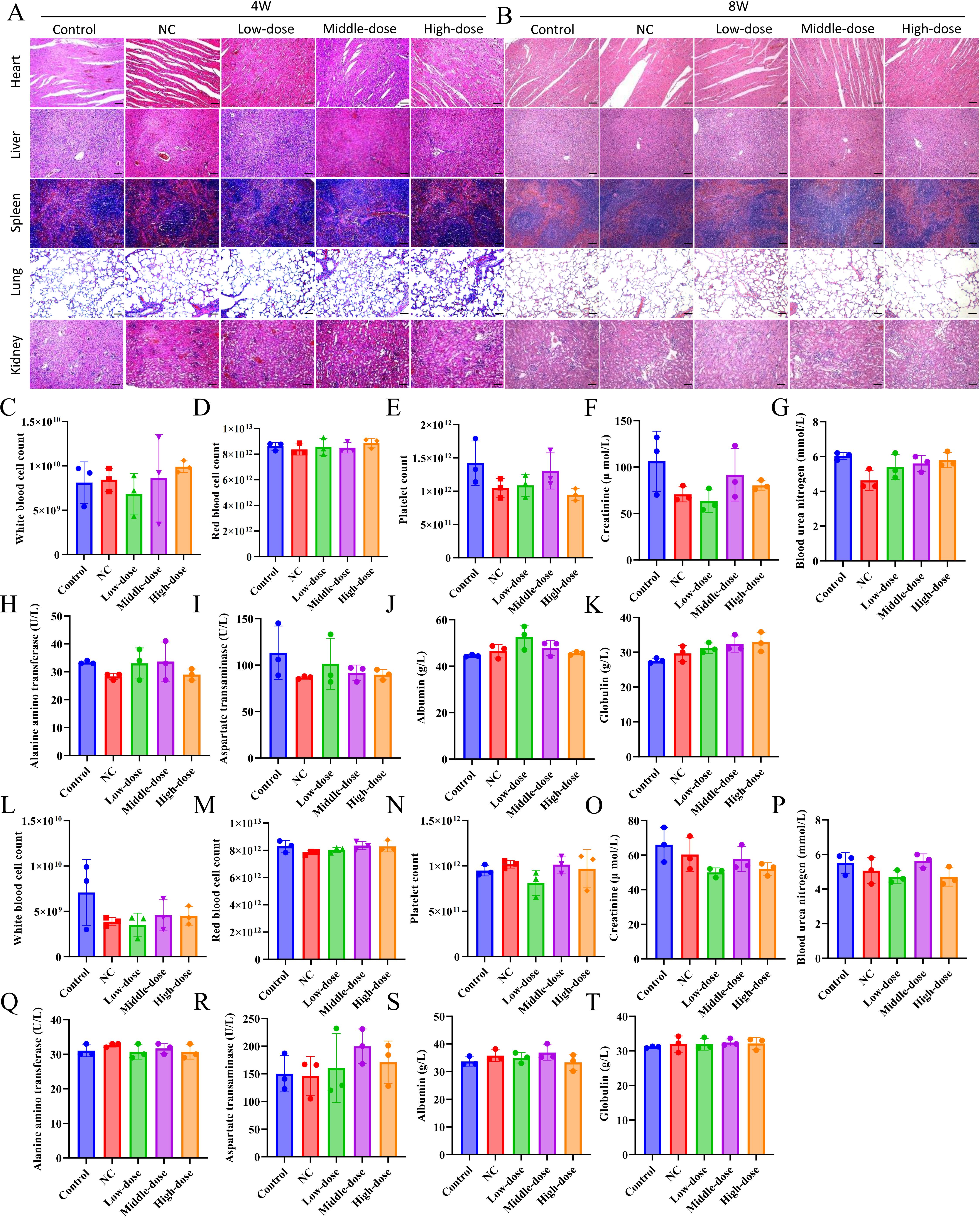


**Figure S9. Veriﬁcation of the biological safety of DMN/PLGA@Dia in vivo.** (A-B) Typical H&E staining images of heart, liver, spleen, lung and kidney in different groups at 4 weeks and 8 weeks. Scale bars = 100 μm (C-K) Blood biochemistry tests at 4 weeks postoperatively, including blood routine test (C-E), renal function tests (F-G) and liver function tests (H-K) (n=3). (L-T) Blood biochemistry tests at 8 weeks postoperatively, including blood tests (L-N), renal function tests (O-P) and liver function tests (Q-T) (n=3)


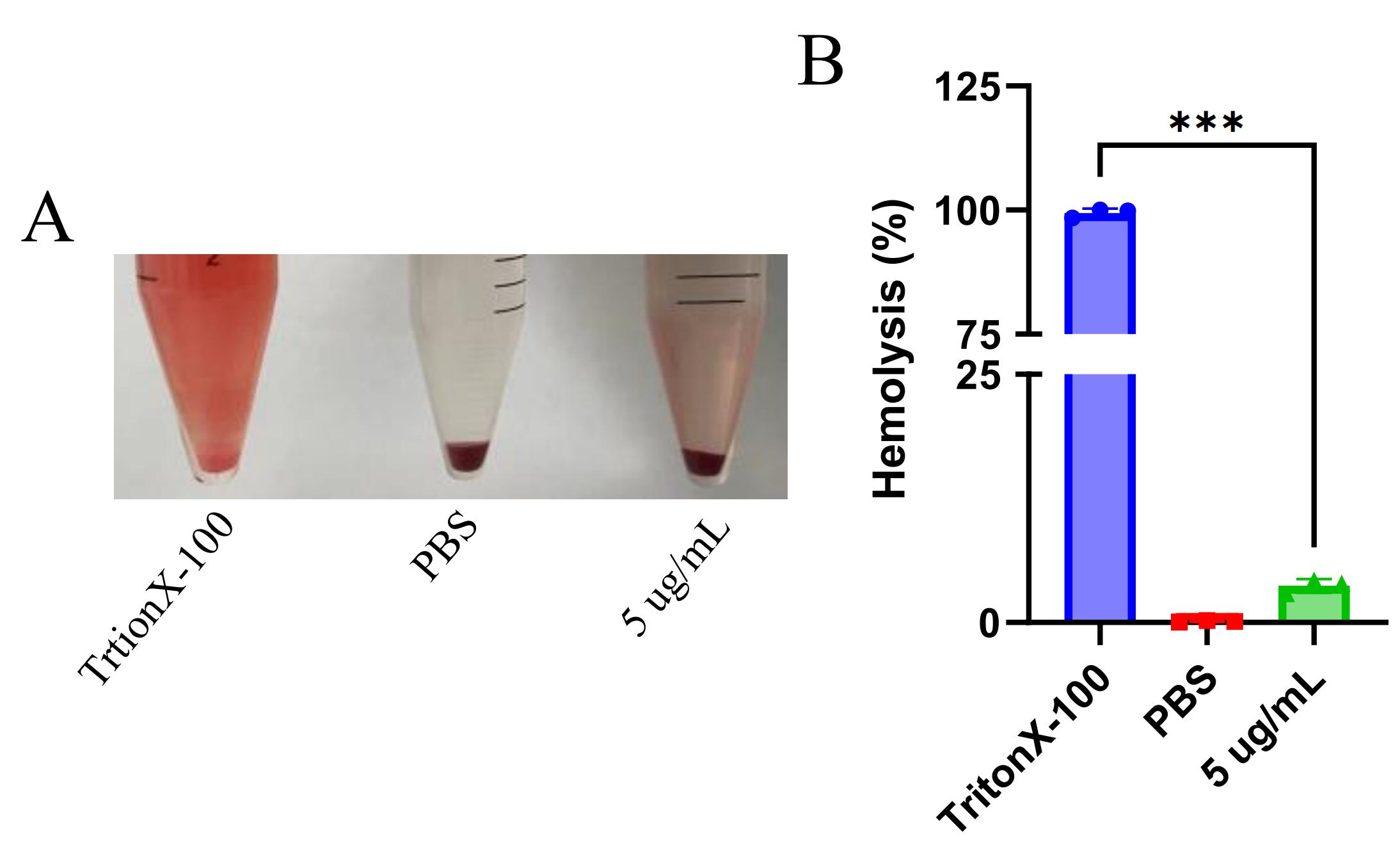


**Figure S10. Hemolysis analysis of nanoparticles.** (A) Typical images of hemolysis analysis. (B) Quantitative analysis of hemolysis rate induced by nanoparticles (n=3). *** P<0.001.
